# Dataset on the diversity of helminth parasites of freshwater fish in the headwaters of the Coatzacoalcos river, in Oaxaca, Mexico

**DOI:** 10.1016/j.dib.2020.106191

**Published:** 2020-08-19

**Authors:** Guillermo Salgado-Maldonado, Juan Manuel Caspeta-Mandujano, Emilio Martínez-Ramírez, Jesús Montoya-Mendoza, Edgar F. Mendoza-Franco

**Affiliations:** a Instituto de Biología, Laboratorio de Helmintología, Universidad Nacional Autónoma de México, Ciudad Universitaria, Coyoacán CP 04510 Ciudad de México, Mexico; b Facultad de Ciencias Biológicas, Laboratorio de Parasitología de Animales Silvestres, Universidad Autónoma del Estado de Morelos, Cuernavaca, Morelos, Mexico; c Departamento de Investigación, Centro Interdisciplinario de Investigación para el Desarrollo Integral Regional, Unidad Oaxaca, Instituto Politécnico Nacional (CIDIIR Oaxaca IPN), área de Acuacultura. Calle Hornos N° 1003, Municipio Santa Cruz Xoxocotlán, C. P. 71230, Oaxaca, Mexico; d Laboratorio de Investigación Acuícola Aplicada, Tecnológico Nacional de México, Instituto Tecnológico de Boca del Río, Km 12 Carretera Veracruz-Córdoba, CP, Boca del Río 94290, Veracruz, Mexico; e Instituto de Ecología, Pesquerías y Oceanografía del Golfo de México (EPOMEX), Universidad Autónoma de Campeche, San Francisco de Campeche, Campeche, Mexico

**Keywords:** Platyhelminthes, Nematoda, Acanthocephala, Monogenea, Trematoda, Cestoda, Metacercariae, geographical distribution

## Abstract

The data presented in this article are related to the research article entitled “***Diversity of helminth parasites of freshwater fish in the headwaters of the Coatzacoalcos river, in Oaxaca, Mexico***” published in International Journal for Parasitolology: Parasites and Wildlife. This dataset document the diversity of helminth parasites found in 25 fish species from 8 families from rivers in the headwaters of the Coatzacoalcos river basin at the border between Oaxaca and Veracruz states, Isthmus of Tehuantepec zone, southeastern Mexico, in the northernmost end of Central America. We record here 48 species, 44 genera and 29 helminth families. Most of the helminth species recorded in this area has also been collected from Central American bodies of freshwater south of Mexico. The material in this Data in Brief paper comprised the raw data on the abundance distribution of each helminth taxa recorded in each of the host and location; i. e. the number of helminth individuals of each helminth taxa found in each one individual fish from each species from each of the localities sampled. The data set is contained in one text-table matrix per fish-host, date of collection and locality of helminth taxa (lines) per fish host species (columns).

**Specifications Table**

**Table:** 

Subject area	Biology; Animal science and Zoology.
Specific subject area	Platyhelminthes, Nematoda and Acanthocephala. Helminth ecto- and endo-parasites of tropical freshwater fish of northern end of Central America.
Type of data	Table.
How data were acquired	Microscope, survey. Each fish was examined under a stereo microscope in Petri dishes with river water for external examination, and with 0.6% saline solution for internal organs. External examination included the skin, scales, mouth, gill cavity, anus, and fins of each host; while internal examination included the brain, gut, mesenteries, kidneys, liver, gall bladder and muscles. We collected data on the number of species (species richness) and abundance distribution of helminths (number of individuals of each species).
Data format	Raw numbers in matrices of localities and fish-individual (lines) vs characteristics (location coordinates and altitude; host length, weigh and sex), and helminth parasite taxa (columns) recorded in each one fish host individually. A matrix per each one of 25 fish species.
Description of data collection	We examined 410 freshwater fishes from 25 species and eight families during March and April 2009; at seven sites in the headwaters of the Coatzacoalcos river basin. Fish were collected using electrofishing device, transferred to the laboratory and kept alive in aerated containers until they were examined for helminths, performed within 8 hours of capture. Each fish was measured (total and standard length; maximum deep) and examined under a stereo microscope in Petri dishes with river water (for external organs) or saline 0.6% solution (for internal organs inspection). Skin, scales, mouth, gill cavity, anus, and fins, as well as all internal organs and tissues except the blood and bones, of each host were examined. Fish were euthanized and the gill arches were removed, separated from the gill cavity and evaluated individually. After, internal organs were excised and examined for helminths under stereomicroscope.
Parameters for data collection	The platyhelminths (monogenea and digenea) and the acanthocephalan found were fixed in 4% hot formaldehyde, stained with Mayer's paracarmine or Gomori´s triple stain and mounted whole on Canada balsam, to made permanent slides for microscopical examination. Nematodes were fixed also in 4% formalin and studied in nonpermanent slides with glycerin. Taxonomic identification was performed based on morphometric analysis of the specimens. A total of 48 helminth species are documented from 44 genera and 29 families.
Data source location	MEXICO (Isthmus of Tehuantepec zone, southeastern of the country) Northermost end of Central America. Seven sample locations situated on the upper Coatzacoalcos river: **1**. El Platanillo river, tributary to Del Sol river (municipality Santo Domingo Petapa), coordinates 16.951111, -95.244167, altitude 416 m; **2**. Río Grande (El Barrio), 16.792167, -95.016083, 220 m; **3**. Río Negro (Santa María Chimalapa), 16.898528, -94.693694, 166 m; **4**. Río Modelo (Santa María Chimalapa), 17.134778, -94.745000, 115 m; **5**. Río Pánfilo (Matías Romero, Oaxaca), 17.083639, -94.873944, 60 m; **6**. Río Jaltepec (Jesús Carranza, Veracruz), 17.388444, -95.056111, 40 m; **7**. Río Escondido (Paraje San Francisco El Vado, Agencia Municipal San Francisco El Vado, Santa María Chimalapa), 17.091083, -94.751694, 83 m. Note all sites in Oaxaca state, except # 6, which is in Veracruz state.
Data accessibility	Data provided within this article.
Related research article	Salgado-Maldonado, G., Caspeta-Mandujano, J. M., Martínez-Ramírez, E., Montoya-Mendoza, J., and Mendoza-Franco, E. F., **Diversity of helminth parasites of freshwater fish in the headwaters of the Coatzacoalcos river, Oaxaca, Mexico**. International Journal for Parasitology: Parasites and Wildlife, 12: 142 - 149. doi.org/10.1016/j.ijppaw.2020.05.008

## Value of the Data

• These data are essential for phylogenetical and ecological hypothesis planing and further biogeographical studies; will assist to examine spatial variation in community structure of helminth parasites of freshwater fishes; may also assist to compare patterns of structure of assemblage vs appropriate null models; can be useful to compare population or community characteristics i. e. richness, densities, of tropical assemblages vs temperate or other regions.
• These kinds of data could be useful for general biologists, biogeographers, ecologists and parasitologits. Also for aquaculturists and veterinarians involved in aquatic organism especially fish management and production; as well as regulatory agencies and stakeholders who seek to protect the public and their goods or values by limiting the adverse environmental impacts of development.
• Parasite species knowledge of fishes and other aquatic organisms can be used to constraint controlled production of aquatic organisms or exploitation of natural resources. Likewise, environmental impact processes would take in account the likelihood that any aquacultural development will affect natural populations, native species or biodiversity in general, for example by accidentally introducing exotic, undesiderable alien species of parasites to the natural populations of fishes (see [1]; Salgado-Maldonado and Rubio-Godoy, 2015, [2] for examples).
• These data could be used for providing an assessment of human impacts on the environment, or to generate data utile for a public awareness of conservation objectives. For example, host parasite systems knowledge can be used to indicate changes in the status of biodiversity [3]. As well these data could support to explore characteristics of the structure of parasite assemblages as nestedness or patterns of decay of similarity with distance.

## 1 Data Description

Table 1 contains the list of the fish species and the number of individual fish examined ordered alphabetically by fish family. Common names of the fish species and locations of collection are also referred in this table.

**Table 1:** Fish families and species, common names, localities (RJ: Jaltepec River, EP El Platanillo River; RE Escondido River; RN Negro River; RG Grande River; RM Modelo River; RP Pánfilo River), dates (Ap, April, Ma, March 2009) and number of hosts examined from the headwaters of the Coatzacoalcos river, Mexico.

	Common names	Locality	No. hosts examined
CHARACIDAE			
*Astyanax aeneus* (Günther, 1860) [referred as *Astyanax finitimus* (Bocourt, 1868) by Schmitter-Soto, 2017]	Platilla, pepesca, Banded tetra	EP/Ma	19
		RN/Ma	24
		RG/Ma	1
		RP/Ap	20
		RJ/Ap	12
CICHLIDAE			
*Parachromis friedrichsthalii* (Heckel, 1840)	Yellowjacket cichlid	RJ/Ap	1
*Paraneetroplus bulleri* Regan, 1905	Mojarra, Corrientero	EP/Ma	2
		RE/Ap	16
*Theraps irregularis* Günther, 1862	Arroyo cichlid	RN/Ma	6
*Thorichthys callolepis* (Regan, 1904)	Mojarra de San Domingo	RN/Ma	30
		RJ/Ap	7
*Thorichthys helleri* (Steindachner, 1864)	Mojarra amarilla, Yellow cichlid	RG/Ma	3
		RM/Ma	8
*Thorichthys maculipinnis* (Steindachner, 1864)	Mojarra	RE/Ap	1
*Trichromis salvini* (Günther, 1862)	Mojarra, Yellow belly cichlid	RN/Ma	9
		RG/Ma	1
		RM/Ma	1
		RP/Ap	3
		RE/Ap	3
		RJ/Ap	9
*Vieja guttulata* (Günther, 1864)	Mojarra de Amatitlán, Amatitlán cichlid	EP/Ma	24
		RN/Ma	29
		RE/Ap	10
		RJ/Ap	6
*Vieja regani* (Miller, 1974)	Mojarra pinta, mojarra de Almoloya, Almoloya cichlid	RG/Ma	5
		RM/Ma	5
ELEOTRIDAE			
*Gobiomorus dormitor* Lacepède, 1800	Guavina, Bigmouth sleeper	RN/Ma	6
		RM/Ma	4
		RP/Ap	4
		RE/Ap	1
		RJ/Ap	1
GOBIIDAE			
*Awaous banana* (Valenciennes, 1837)	Gobio de río	RN/Ma	8
		RM/Ma	1
HEPTAPTERIDAE			
*Rhamdia guatemalensis* (Günther, 1864)	Juile, Bagre, Guatemalan chulin	RG/Ma	1
		RN/Ma	4
		RP/Ap	5
*Rhamdia laticauda* (Kner, 1858)	Bagre, Oaxaca catfish, Filespine chulín	RE/Ap	6
MUGILIDAE			
*Agonostomus monticola* (Bancroft, 1834)	Lisa de río, Mulet	RN/Ma	1
		RP/Ap	2
		RE/Ap	2
POECILIIDAE			
*Poecilia mexicana* Steindachner, 1863	Shortfin molly	RE/Ap	13
*Poecilia sphenops* Valenciennes, 1846	Guppi, Molly	RM/Ma	2
		RN/Ma	5
		RG/Ma	5
		RJ/Ap	10
*Poeciliopsis gracilis* (Heckel, 1848)	Porthole livebearer	RG/Ma	4
		RN/Ma	1
		RE/Ap	1
*Priapella intermedia* Álvarez and Carranza, 1952	Guayacón de los Chimalapas	RG/Ma	1
*Pseudoxiphophorus bimaculatus* (Heckel, 1848)	Guatopote manchado, Twospot livebearer	EP/Ma	4
		RN/Ma	1
		RP/Ap	8
*Xiphophorus clemenciae* Álvarez, 1959	Espadita, Yellow swordtail	RG/Ma	3
		RM/Ma	2
		RN/Ma	4
		RE/Ap	9
*Xiphophorus mixei* Kallman, Walter, Morizot and Kazianis, 2004	Mixe swordtail	EP/Ma	5
*Xiphophorus monticolus* Kallman, Walter, Morizot and Kazianis, 2004	Southern mountain swordtail	EP/Ma	9
*Xiphophorus helerii* Heckel, 1848	Espadita, Green swordtail	RJ/Ap	2
SYNBRANCHIDAE			
*Ophisternon aenigmaticum* Rosen and Greenwood, 1976	Falsa anguila, Fatlips swamp eel	EP/Ma	5
		RG/Ma	1
		RM/Ma	1
		RN/Ma	3
		RE/Ap	7
		RJ/Ap	3

Table 2 is a list of fish host – parasite associations, including the tissues or organs of the fish from which parasites were collected, and the localities and date of collection of helminth parasites collected from the 25 fish species examined from upper Coatzacoalcos river, Oaxaca, Mexico. Helminth parasites are ordered by Platyhelminthes (Monogenea, Trematoda, Cestoda), Acanthocephala and Nematoda (adults first, then larval forms).

**Table 2:** Parasite – host associations, localities (RJ: Jaltepec River, EP El Platanillo River; RE Escondido River; RN Negro River; RG Grande River; RM Modelo River; RP Pánfilo River), and date of collection (Ap, April or Ma, March 2009), of helminth parasites collected from 25 fish species from upper Coatzacoalcos river, Oaxaca, Mexico

HELMINTH FISH HOST	SITE	LOCALITY, Date
**MONOGENEA**		
*Aphanoblastella travassosi* (Price, 1938)		
*Rhamdia guatemalensis*	Gills	RN, Ma
*Rhamdia laticauda*	Gills	RE, Ap
*Guavinella tropica* Mendoza-Franco, Scholz and Cabañas-Carranza, 2003		
*Gobiomorus dormitor*	Gills	RN, RM, Ma; RE, Ap
*Gyrodactylus* sp.		
*Poecilia mexicana*	Fins	RE, Ap
*Thorichthys callolepis*	Fins	RN, Ma
*Vieja guttulata*	Gills	RN, Ma
*Salsuginus* sp.		
*Xiphophorus monticolus*	Gills	EP, Ma
*Sciadicleithrum* sp.		
*Paraneetroplus bulleri*	Gills	RE, Ap
*Thorichthys callolepis*	Gills	RN, Ma
*Vieja guttulata*	Gills	EP, Ma; RJ, Ap
*"Urocleidoides"* cf. *strombicirrus* (Price and Bussing, 1967)		
*Astyanax aeneus*	Gills	EP, RN, Ma; RP, Ap
**TREMATODA**		
*Auriculostoma astyanace* Scholz, Aguirre-Macedo and Choudhury, 2004		
*Astyanax aeneus*	Intestine	RG, Ma; RJ, Ap
*Crassicutis cichlasomae* Manter, 1936		
*Parachromis friedrichsthalii*	Intestine	RJ, Ap
*Paraneetroplus bulleri*	Intestine	RE, Ap
*Thorichthys helleri*	Intestine	RM, Ma
*Trichromis salvini*	Intestine	RM, Ma; RP, RE, RJ, Ap;
*Vieja regain*	Intestine	RM, Ma
*Creptotrema agonostomi* Salgado-Maldonado, Cabañas-Carranza and Caspeta-Mandujano, 1998		
*Agonostomus monticola*	Intestine	RN, Ma; RP, RE, Ap
*Genarchella astyanctis* (Watson, 1976)		
*Astyanax aeneus*	Intestine	RP, RJ, Ap
*Genarchella isabellae* (Lamothe-Argumedo, 1977)		
*Gobiomorus dormitor*	Intestine	RM, Ma
*Thorichthys helleri*	Intestine	RM, Ma
*Vieja guttulata*	Stomach	EP, RN, Ma
*Vieja regain*	Stomach	RM, Ma
*Magnivitellinum* cf. *simplex* Kloss, 1966		
*Astyanax aeneus*	Intestine	RP, Ap
*Paracreptotrematoides* cf. *heterandriae* (Salgado-Maldonado, Caspeta-Mandujano and Vázquez, 2012)		
*Pseudoxiphophorus bimaculatus*	Intestine	RP, Ap
*Saccocoelioides* cf. *sogandaresi* Lumsden, 1963		
*Agonostomus montícola*	Intestine	RP, Ap
*Poecilia sphenops*	Intestine	RG, Ma
*Poeciliopsis gracilis*	Intestine	RG, Ma
*Xiphophorus clemenciae*	Intestine	RG, Ma
*Wallinia anindoi* Hernández-Mena, Pinacho-Pinacho, García-Varela, Mendoza-Garfías and Pérez Ponce de León, 2019		
*Astyanax aeneus*	Intestine and intestinal caeca	RN, Ma; RJ, RP, Ap
**METACERCARIAE**		
*Apharyngostrigea* sp.		
*Astyanax aeneus*	Mesentery, Gall bladder	RN, Ma; RJ Ap
*Ascocotyle* (*Phagicola*) *diminuta* (Stunkard and Haviland, 1924)		
*Poecilia sphenops*	Gills	RG, Ma
*Centrocestus formosanus* (Nishigori, 1924)		
*Astyanax aeneus*	Gills	RN, Ma
*Gobiomorus dormitor*	Gills	RN, Ma
*Pseudoxiphophorus bimaculatus*	Gills	RP, Ap
*Thorichthys callolepis*	Gills	RN, Ma
*Xiphophorus clemenciae*	Gills	RN, Ma
*Cladocystis* cf. *trifolium* (Braun, 1901)		
*Thorichthys helleri*	Intestine	RM, Ma
*Trichromis salvini*	Mesentery	RG, Ma
*Clinostomum* sp.		
*Astyanax aeneus*	Gills, fins, mesentery	EP, Ma
*Rhamdia guatemalensis*	Mesentery, fins, gill cavity, mesentery	RN, Ma; RP, Ap
*Rhamdia laticauda*	Fins	RE, Ap
*Thorichthys callolepis*	Fins, skin, mouth, mesentery	RN, Ma
*Vieja guttulata*	Gill cavity	RN, Ma
*Crocodilicola pseudostoma* (Willemoes-Suhm, 1870)		
*Rhamdia guatemalensis*	Intestine	RP, Ap
*Diplostomum* sp.		
*Poecilia sphenops*	Eyes	RJ, Ap
*Thorichthys callolepis*	Eyes, gills	RN; Ma; Rj, Ap
*Trichromis salvini*	Eyes	RN, Ma; RJ, Ap
*Vieja guttulata*	Eyes, brain, mesentery	RN, Ma; RJ, Ap
*Posthodiplostomum* sp.		
*Parachromis friedrichsthalii*	Mesentery	RJ, Ap
*Paraneetroplus bulleri*	Muscle, mesentery	RE, Ap
*Poecilia sphenops*	Mesentery	RG, Ma; Rj, Ap
*Trichromis salvini*	Muscle, eyes, mesentery	RJ, Ap
*Vieja guttulata*	Muscle, mesentery	RN, Ma
	Muscle	RJ, Ap
*Vieja regain*	Gills	RG, Ma
*Tylodelphys* sp.		
*Parachromis friedrichsthalii*	Mesentery	RJ, Ap
*Trichromis salvini*	Mesentery	RJ, Ap
*Uvulifer* cf. *ambloplitis* (Hughes, 1927)		
*Astyanax aeneus*	Skin, fins, muscle	EP, Ma
*Paraneetroplus bulleri*	Muscle	EP, Ma
*Priapella intermedia*	Fins	RG, Ma
*Xiphophorus clemenciae*	Fins	RG, Ma
**CESTODA**		
*Cichlidocestus* sp.		
*Thorichthys callolepis*	Intestine	RN, Ma
*Vieja guttulata*	Intestine	RN, Ma
*Schyzocotyle acheilognathi* (Yamaguti, 1934)		
*Vieja guttulata*	Intestine	RJ, Ap
**METACESTODE**		
*Glossocercus* sp.		
*Poecilia sphenops*	Liver	RJ, Ap
**ACANTHOCEPHALA**		
*Neoechinorhynchus chimalapasensis* Salgado-Maldonado, Caspeta-Mandujano and Martínez-Ramírez, 2010		
*Awaous banana*	Intestine	RN, Ma
**NEMATODA**		
*Atractis vidali* González-Solís and Moravec, 2002		
*Vieja guttulata*	Intestine	RN, Ma
Capillariidae gen. sp.		
*Astyanax aeneus*	Stomach	RP, Ap
*Cucullanus angeli* Cabañas-Carranza and Caspeta-Mandujano, 2007		
*Vieja guttulata*	Intestine	EP, RN, Ma
*Cucullanus mexicanus* Caspeta-Mandujano, Moravec and Aguilar-Aguilar, 2000		
*Rhamdia guatemalensis*	Mesentery	RP, Ap
*Cucullanus* sp.		
*Gobiomorus dormitor*	Intestine	RE, Ap
*Paraneetroplus bulleri*	Intestine	RE, Ap
*Thorichthys helleri*	Intestine	RM, Ma
*Vieja guttulata*	Intestine	RE, Ap
*Dichelyne mexicanus* Caspeta-Mandujano, Moravec and Salgado-Maldonado, 1999		
*Agonostomus monticola*	Intestine	RP, Ap
*Parcapillaria teixeirafreitasi* (Caballero-Rodríguez, 1971)		
*Gobiomorus dormitor*	Stomach	RE, Ap; RN, Ma
Philometridae gen. sp.		
*Ophisternon aenigmaticum*	Skin	EP, RN, Ma
*Paraneetroplus bulleri*	Body cavity	RE, Ap
*Theraps irregularis*	Muscle	RN, Ma
*Procamallanus* (*Spirocamallanus*) *rebecae* (Andrade-Salas, Pineda-López and García-Magaña, 1994)		
*Thorichthys helleri*	Intestine	RG, RM, Ma
*Thorichthys maculipinnis*	Intestine	RE, Ap
*Pseudocapillaria* (*Icthyocapillaria*) *ophisterni* Moravec, Salgado-Maldonado and Jiménez-García, 2000		
*Ophisternon aenigmaticum*	Mesentery	RE, Ap
*Raillietnema kritscheri* Moravec, Salgado-Maldonado and Pineda-López, 1993		
*Paraneetroplus bulleri*	Intestine	RE, Ap
*Thorichthys helleri*	Intestine	RM, Ma
*Trichromis salvini*	Intestine	RJ, Ap
*Vieja guttulata*	Intestine	RN, Ma; RE, Ap
*Vieja regain*	Intestine	RG, Ma
*Rhabdochona kidderi* Pearse, 1936		
*Paraneetroplus bulleri*	Intestine	RE, Ap
*Rhamdia laticauda*	Intestine	RE, Ap
*Vieja guttulate*	Intestine	EP, Ma; RE, Ap
*Vieja regain*	Intestine	RM, Ma
*Rhabdochona* sp.		
*Theraps irregularis*	Intestine	RN, Ma
*Spinitectus mexicanus* Caspeta-Mandujano, Moravec and Salgado-Maldonado, 2000		
*Pseudoxiphophorus bimaculatus*	Intestine	RP, Ap
**LARVAL NEMATODES**		
Acuariidae gen. sp.		
*Astyanax aeneus*	Intestine	EP, Ma
*Paraneetroplus bulleri*	Mesentery	EP, Ma
*Trichromis salvini*	Mesentery	RG, Ma
*Xiphophorus mixei*	Muscle	EP, Ma
*Contracaecum* sp.		
*Astyanax aeneus*	Liver	RN, Ma; RP, Ap
*Awaous banana*	Mesentery	RN, Ma
*Gobiomorus dormitor*	Intestine, liver, muscle, mesentery	RM, RN, Ma; RE, RP, Ap
*Ophisternon aenigmaticum*	Body cavity, Mesentery	RG, RM, RN, Ma; RJ, RP, Ap
*Paraneetroplus bulleri*	Liver, mesentery	RE, Ap
*Rhamdia guatemalensis*	Mesentery	RG, Ma; RP, Ap
*Thorichthys callolepis*	Liver	RJ, Ap
*Thorichthys helleri*	Liver	RM, Ma
*Vieja guttulate*	Intestine, mesentery	RE, RJ, Ap
*Vieja regani*	Mesentery	RG, Ma
*Xiphophorus hellerii*	Mesentery	RJ, Ap
*Falcaustra* sp.		
*Gobiomorus dormitor*	Intestine	RN, Ma
*Poecilia mexicana*	Intestine	RE, Ap
*Hysterothylacium cenotae* (Pearse, 1936)		
*Vieja guttulate*	Intestine	RN, Ma
*Rhabdochona* sp. larvae or female unidentifiable to species		
*Gobiomorus dormitor*	Intestine	RM, Ma; RP, Ap
*Ophisternon aenigmaticum*	Intestine	RE, Ap
*Thorichthys helleri*	Intestine	RM, Ma
*Vieja guttulate*	Intestine	RN, Ma
*Spiroxys* sp.		
*Astyanax aeneus*	Mesentery	RJ, Ap; RN, Ma
*Gobiomorus dormitor*	Mesentery	RN, Ma
*Poecilia mexicana*	Mesentery	RE, Ap
*Thorichthys callolepis*	Mesentery	RN, Ma
*Xiphophorus clemenciae*	Mesentery	RG, Ma

Table 3 list the abbreviations to the scientific names of the taxa of helminth parasites used in Supplementary Table 4.

**Table 3:** Abbreviations to scientific names of helminth parasite taxa referred to in Supplementary Table 4.

*Aas*	*Auriculostoma astyanace*	*Gyr*	*Gyrodactylus* sp.
*Acu*	Acuariidae	*Hce*	*Hysterothylacium cenotae* (larvae)
*Adi*	*Ascocotyle* (*Phagicola*) *diminuta*	*Msi*	*Magnivitellinum* cf*. Simplex*
*Aph*	*Apharyngostrigea* sp.	*Nch*	*Neoechinorhynchus chimalapasensis*
*Atr*	*Aphanoblastella travassosi*	*Phe*	*Paracreptotrematoides* cf. *heterandriae*
*Avi*	*Actractis vidali*	*Phi*	Phillometridae gen. sp.
*Cag*	*Creptotrema agonostomi*	*Pmi*	*Posthodiplostomum cf. minimum*
*Can*	*Cucullanus angeli*	*Pop*	*Pseudocapillaria ophistherni*
*Cap*	*Capillaria* sp.	*Pos*	*Posthodiplostomum* sp.
*Cci*	*Crassicutis cichlasomae*	Pre	*Procamallanus rebecae*
*Cfo*	*Centrocestus formosanus*	*Pte*	*Paracapillaria teixeirafreitasi*
*Cic*	*Cichidocestus* sp.	*Rha*	*Rhabdochona* sp.
*Cli*	*Clinostomum* sp.	*Rki*	*Rhabdochona kidderi*
*Cme*	*Cucullanus mexicanus*	*Rkr*	*Raillietnema kritscheri*
*Con*	*Contracaecum* sp.	*Sac*	*Schyzocotyle acheilognathi*
*Cps*	*Crocodilicola pseudostoma*	*Sci*	*Sciadicleithrum* sp.
*Ctr*	*Cladocystis trifolium*	*Sme*	*Spinitectus mexicanus*
*Cuc*	*Cucullanus* sp.	*Spi*	*Spiroxys* sp.
*Dip*	*Diplostomum* sp.	*Sso*	*Saccocoelioides* cf. *sogandaresi*
*Dme*	*Dichelyne mexicanus*	*Tyl*	*Tylodelphis*
*Fal*	*Falcaustra* sp.	*Uam*	*Uvulifer ambloplitis*
*Gas*	*Genarchella astyanctis*	*Ust*	*Urocleidoides* cf. *strombicirrus*
*Gis*	*Genarchella isabellae*	*Wan*	*Walliniea anindoi*
*Glo*	*Glossocercus* sp.		
*Gtr*	*Guavinella tropica*		

Supplementary Table 4. document the raw data on helminth parasites of 25 fish species from the Headwaters of Coatzacoalcos river, Mexico. One matrix for each fish species ordered alphabetically by fish families (see Table 1). Data include the name of the locality, coordinates in decimal degrees, altitude meters above sea level, date of collection, host number in the author's field notes, host’ sex; host’ measurements documented for each one fish examined; and the raw number of helminth parasites recorded from each fish host.

## 2 Experimental design, materials and methods

We gathered data from a total of 410 freshwater fish from 25 species and eight families during March and April 2009. Fishes were caugh at the upper reaches of the Coatzacoalcos River basin. The area of study is located ∼300 km from the mouth of the Coatzacoalcos river in the Gulf of Mexico. We examined from 1 to 30 individuals of every available fish species from each of seven locations. Sample locations were chosen as follows: **1**. El Platanillo river, tributary to Del Sol river (municipality Santo Domingo Petapa), coordinates 16.951111, -95.244167, altitude 416 m; **2**. Río Grande (El Barrio), 16.792167, -95.016083, 220 m; **3**. Río Negro (Santa María Chimalapa), 16.898528, -94.693694, 166 m; **4**. Río Modelo (Santa María Chimalapa), 17.134778, -94.745000, 115 m; **5**. Río Pánfilo (Matías Romero, Oaxaca), 17.083639, -94.873944, 60 m; **6**. Río Jaltepec (Jesús Carranza, Veracruz), 17.388444, -95.056111, 40 m; **7**. Río Escondido (Paraje San Francisco El Vado, Agencia Municipal Río Escondido, Santa María Chimalapa), 17.091083, -94.751694, 103 m. Note all sites in Oaxaca state, except # 6, which is in Veracruz state, Mexico. At each locality, fish were captured using nets or electrofishing device. Live fish were brought to the laboratory and examined within 8 h of capture using standard procedures. Briefly, all the external surfaces, víscera, and musculature of each fish host were examined under a stereomicroscope, and all the helminths encountered in each fish were counted. Two kinds of data were collected from each individual fish: the number of helminth taxa (species richness) in each fish and the number of helminth individuals per helminth taxa (the abundance distribution). All helminths found were isolated and counted, and then fixed in 4% hot formaldehyde (cestodes, monogeneans and adult digeneans, as well as larvae of digeneans and nematodes). Some monogeneans were fixed with ammonium picrate [4] and mounted unstained in Gray–Wess medium [5], for analysis of sclerotized structures. Acanthocephalans were placed in distilled water, refrigerated overnight (6–12 h) to evert the proboscis, and then fixed in hot 10% formalin. Digeneans, monogeneans, cestodes, and acanthocephalans used for morphological examination of whole mounts, were stained with either Mayer's paracarmine or Gomori´s triple stain dehydrated using a graded alcohol series, cleared in methyl salicylate, and and mounted whole in Canada balsam. Nematodes were cleared in glycerine for light microscopy and stored in 70% ethanol. Taxonomic identification was performed based on morphometric analysis of the specimens [6].

## Ethics statement

Fish were euthanized and the branchial arches were removed, separated from the brachial cavity and evaluated individually (protocol for the use of fish in research based on the NORM – 019 – STPS – 1993 established by the Instituto de Ecología, Pesquerías y Oceanografía del Golfo de México EPOMEX, Campeche, Mexico; specimens collected under the Cartilla Nacional de Colector Científico FAUT-0105 issued by the Secretaría del Medio Ambiente y Recursos Naturales [SEMARNAT] to GSM).

## Declaration of Competing Interest

The authors declare that they have no known competing financial interests or personal relationships that could have appeared to influence the work reported in this paper.

## Appendix Supplementary materials

Image, application 1

Image, application 1

## Footnotes

Supplementary material associated with this article can be found, in the online version, at doi:10.1016/j.dib.2020.106191.
