# Supplementary material for: Dataset on the diversity of helminth parasites of freshwater fish in the headwaters of the Coatzacoalcos river, in Oaxaca, Mexico
Source: Data Brief. 2020 Aug 19;32:106191. doi: 10.1016/j.dib.2020.106191 (PMC7476224; doi:10.1016/j.dib.2020.106191)
Supplement: Supplementary file 2 [file mmc2.docx]

*Table 4. Raw data on helminth parasites of 25 fish species from the Headwaters of Coatzacoalcos river, Mexico. One matrix for each fish species ordered alphabetically by fish families (see Table 1). Data include the name of the locality, coordinates in decimal degrees, Alt = altitude meters above sea level, date of collection Ma = March, Ap, April 2009, Host# = host number in the author’s field notes, sex = host’ sex, M = Male, F = Female; host’ measurements including Tl = total length mm, Sl = standard length mm, Md = Maximum deep mm, and We = weight gr, documented for each one fish examined; and the raw number of helminth parasites recorded from each fish host (see abbreviations for the helminth taxa in Table 2). NA = missing data, no parasites.*

**CHARACIDAE**

| **Table_4A_*Astyanax_aeneus*** | |  |  |  |  |  |  |  |  |  |  |  |  |  |  |  |  |  |  |  |  |  |  |  |
| --- | --- | --- | --- | --- | --- | --- | --- | --- | --- | --- | --- | --- | --- | --- | --- | --- | --- | --- | --- | --- | --- | --- | --- | --- |
| Locality | Latitude | Longitude | Alt | Date | Host # | sex | Tl | Sl | Md | We | *Ust* | *Aas* | *Gas* | *Msi* | *Wan* | *Aph* | *Cfo* | *Cli* | *Uam* | *Cap* | *Rkr* | Acu | *Con* | *Spi* |
| Rio Grande | 16.79217 | -95.01608 | 451 | Ma | 10931 | H | 93 | 74 | 23 | 9.6 | NA | 1 | NA | NA | NA | NA | NA | NA | NA | NA | NA | NA | NA | NA |
| Rio Negro | 16.89853 | -94.69369 | 421 | Ma | 10992 | M | 70 | 60 | 20 | 4.3 | NA | NA | NA | NA | NA | NA | NA | NA | NA | NA | NA | NA | NA | NA |
| Rio Negro | 16.89853 | -94.69369 | 421 | Ma | 109104 | H | 110 | 85 | 40 | 20.3 | NA | NA | NA | NA | NA | NA | NA | NA | NA | NA | NA | NA | NA | NA |
| Rio Negro | 16.89853 | -94.69369 | 421 | Ma | 10997 | H | 71 | 61 | 20 | 3.7 | NA | NA | NA | NA | NA | NA | NA | NA | NA | NA | NA | NA | NA | NA |
| Rio Negro | 16.89853 | -94.69369 | 421 | Ma | 109105 | M | 75 | 60 | 24 | 5.8 | 4 | NA | NA | NA | NA | NA | NA | NA | NA | NA | NA | NA | NA | NA |
| Rio Negro | 16.89853 | -94.69369 | 421 | Ma | 10994 | M | 77 | 64 | 23 | 5.2 | NA | NA | NA | NA | 1 | NA | NA | NA | NA | NA | NA | NA | NA | NA |
| Rio Negro | 16.89853 | -94.69369 | 421 | Ma | 109106 | H | 80 | 68 | 23 | 7.4 | NA | NA | NA | NA | NA | NA | NA | NA | NA | NA | NA | NA | NA | NA |
| Rio Negro | 16.89853 | -94.69369 | 421 | Ma | 109118 | M | 72 | 58 | 18 | 4 | NA | NA | NA | NA | NA | NA | NA | NA | NA | NA | NA | NA | 1 | 2 |
| Rio Negro | 16.89853 | -94.69369 | 421 | Ma | 109111 | H | 90 | 75 | 15 | 8.9 | NA | NA | NA | NA | NA | NA | NA | NA | NA | NA | NA | NA | NA | NA |
| Rio Negro | 16.89853 | -94.69369 | 421 | Ma | 10995 | M | 67 | 52 | 17 | 3.3 | 3 | NA | NA | NA | 1 | NA | NA | NA | NA | NA | NA | NA | 2 | 3 |
| Rio Negro | 16.89853 | -94.69369 | 421 | Ma | 10991 | H | 75 | 60 | 18 | 4.9 | 7 | NA | NA | NA | 2 | NA | 1 | NA | NA | NA | NA | NA | NA | NA |
| Rio Negro | 16.89853 | -94.69369 | 421 | Ma | 109113 | J | 90 | 70 | 24 | 8.6 | NA | NA | NA | NA | 11 | NA | NA | NA | NA | NA | NA | NA | NA | NA |
| Rio Negro | 16.89853 | -94.69369 | 421 | Ma | 109107 | J | 70 | 60 | 20 | 4.2 | NA | NA | NA | NA | 1 | NA | NA | NA | NA | NA | NA | NA | NA | NA |
| Rio Negro | 16.89853 | -94.69369 | 421 | Ma | 109100 | H | 100 | 85 | 30 | 13.4 | NA | NA | NA | NA | NA | NA | NA | NA | NA | NA | NA | NA | NA | NA |
| Rio Negro | 16.89853 | -94.69369 | 421 | Ma | 10993 | J | 75 | 64 | 20 | 3.3 | NA | NA | NA | NA | NA | 1 | NA | NA | NA | NA | NA | NA | NA | NA |
| Rio Negro | 16.89853 | -94.69369 | 421 | Ma | 109108 | M | 68 | 59 | 18 | 4.6 | 2 | NA | NA | NA | 1 | NA | NA | NA | NA | NA | NA | NA | NA | NA |
| Rio Negro | 16.89853 | -94.69369 | 421 | Ma | 109103 | M | 73 | 57 | 18 | 4.9 | NA | NA | NA | NA | NA | NA | NA | NA | NA | NA | NA | NA | NA | NA |
| Rio Negro | 16.89853 | -94.69369 | 421 | Ma | 109110 | H | 63 | 50 | 18 | 3.1 | 5 | NA | NA | NA | NA | NA | NA | NA | NA | NA | NA | NA | NA | NA |
| Rio Negro | 16.89853 | -94.69369 | 421 | Ma | 109114 | H | 68 | 54 | 10 | 4 | 2 | NA | NA | NA | 1 | NA | NA | NA | NA | NA | NA | NA | NA | NA |
| Rio Negro | 16.89853 | -94.69369 | 421 | Ma | 10990 | H | 65 | 53 | 19 | 3.3 | 5 | NA | NA | NA | 2 | NA | NA | NA | NA | NA | NA | NA | NA | NA |
| Rio Negro | 16.89853 | -94.69369 | 421 | Ma | 109998 | H | 75 | 60 | 18 | 4.5 | 2 | NA | NA | NA | 4 | NA | NA | NA | NA | NA | NA | NA | NA | NA |
| Rio Negro | 16.89853 | -94.69369 | 421 | Ma | 10999 | M | 94 | 74 | 28 | 9.1 | NA | NA | NA | NA | NA | NA | NA | NA | NA | NA | NA | NA | NA | NA |
| Rio Negro | 16.89853 | -94.69369 | 421 | Ma | 109112 | H | 66 | 52 | 18 | 4.8 | NA | NA | NA | NA | NA | NA | NA | NA | NA | NA | NA | NA | NA | NA |
| Rio Negro | 16.89853 | -94.69369 | 421 | Ma | 109117 | NA | 78 | 56 | 21 | 4.4 | NA | NA | NA | NA | NA | NA | NA | NA | NA | NA | NA | NA | NA | NA |
| Rio Negro | 16.89853 | -94.69369 | 421 | Ma | 109147 | H | 100 | 80 | 26 | 10.5 | NA | NA | NA | NA | NA | NA | NA | NA | NA | NA | NA | NA | NA | NA |
| El Platanillo | 16.95111 | -95.24417 | 346 | Ma | 10902 | H | 80 | 60 | 20 | 6.7 | NA | NA | NA | NA | NA | NA | NA | NA | NA | NA | NA | NA | NA | NA |
| El Platanillo | 16.95111 | -95.24417 | 346 | Ma | 10903 | H | 104 | 82 | 27 | 13 | NA | NA | NA | NA | NA | NA | NA | NA | NA | NA | NA | NA | NA | NA |
| El Platanillo | 16.95111 | -95.24417 | 346 | Ma | 10904 | H | 85 | 65 | 27 | 6.9 | NA | NA | NA | NA | NA | NA | NA | 1 | NA | NA | NA | NA | NA | NA |
| El Platanillo | 16.95111 | -95.24417 | 346 | Ma | 10905 | H | 95 | 80 | 25 | 9.2 | NA | NA | NA | NA | NA | NA | NA | NA | NA | NA | NA | NA | NA | NA |
| El Platanillo | 16.95111 | -95.24417 | 346 | Ma | 10906 | H | 125 | 105 | 40 | 15.3 | NA | NA | NA | NA | NA | NA | NA | 8 | NA | NA | NA | NA | NA | NA |
| El Platanillo | 16.95111 | -95.24417 | 346 | Ma | 10908 | H | 73 | 57 | 21 | 4.7 | NA | NA | NA | NA | NA | NA | NA | NA | NA | NA | NA | NA | NA | NA |
| El Platanillo | 16.95111 | -95.24417 | 346 | Ma | 10909 | H | 89 | 74 | 20 | 8 | 11 | NA | NA | NA | NA | NA | NA | NA | 33 | NA | NA | NA | NA | NA |
| El Platanillo | 16.95111 | -95.24417 | 346 | Ma | 10910 | H | 87 | 72 | 24 | 8.3 | NA | NA | NA | NA | NA | NA | NA | NA | NA | NA | NA | NA | NA | NA |
| El Platanillo | 16.95111 | -95.24417 | 346 | Ma | 10911 | H | 88 | 72 | 21 | 8.3 | NA | NA | NA | NA | NA | NA | NA | 1 | 2 | NA | NA | 1 | NA | NA |
| El Platanillo | 16.95111 | -95.24417 | 346 | Ma | 10912 | H | 75 | 60 | 20 | 5.5 | NA | NA | NA | NA | NA | NA | NA | 4 | NA | NA | NA | NA | NA | NA |
| El Platanillo | 16.95111 | -95.24417 | 346 | Ma | 10913 | H | 100 | 75 | 30 | 9.2 | NA | NA | NA | NA | NA | NA | NA | 3 | NA | NA | NA | NA | NA | NA |
| El Platanillo | 16.95111 | -95.24417 | 346 | Ma | 10914 | H | 62 | 47 | 13 | 1.6 | NA | NA | NA | NA | NA | NA | NA | NA | NA | NA | NA | NA | NA | NA |
| El Platanillo | 16.95111 | -95.24417 | 346 | Ma | 10916 | H | 86 | 60 | 20 | 3.5 | NA | NA | NA | NA | NA | NA | NA | NA | 1 | NA | NA | NA | NA | NA |
| El Platanillo | 16.95111 | -95.24417 | 346 | Ma | 10918 | H | 69 | 57 | 17 | 4.3 | NA | NA | NA | NA | NA | NA | NA | NA | NA | NA | NA | NA | NA | NA |
| El Platanillo | 16.95111 | -95.24417 | 346 | Ma | 10919 | H | 82 | 70 | 18 | 7.2 | NA | NA | NA | NA | NA | NA | NA | NA | 2 | NA | NA | NA | NA | NA |
| El Platanillo | 16.95111 | -95.24417 | 346 | Ma | 10920 | M | 99 | 86 | 35 | 9.9 | NA | NA | NA | NA | NA | NA | NA | NA | NA | NA | NA | NA | NA | NA |
| El Platanillo | 16.95111 | -95.24417 | 346 | Ma | 10922 | H | 87 | 71 | 27 | 7.8 | NA | NA | NA | NA | NA | NA | NA | NA | 1 | NA | NA | NA | NA | NA |
| El Platanillo | 16.95111 | -95.24417 | 346 | Ma | 10923 | M | 72 | 64 | 22 | 4.2 | NA | NA | NA | NA | NA | NA | NA | NA | 1 | NA | NA | NA | NA | NA |
| El Platanillo | 16.95111 | -95.24417 | 346 | Ma | 10926 | H | 70 | 56 | 18 | 4.1 | NA | NA | NA | NA | NA | NA | NA | NA | NA | NA | NA | NA | NA | NA |
| Río Jaltepec | 17.38844 | -95.05611 | 40 | Ap | 209 112 | J | 35 | 25 | 15 | 2.1 | NA | NA | NA | NA | NA | NA | NA | NA | NA | NA | NA | NA | NA | NA |
| Río Jaltepec | 17.38844 | -95.05611 | 40 | Ap | 209113 | H |  |  |  | 3 | NA | 1 | 1 | NA | NA | 1 | NA | NA | NA | NA | NA | NA | NA | 2 |
| Río Jaltepec | 17.38844 | -95.05611 | 40 | Ap | 209114 | M | 80 | 40 | 12 | 1.3 | NA | NA | 1 | NA | NA | NA | NA | NA | NA | NA | NA | NA | NA | NA |
| Río Jaltepec | 17.38844 | -95.05611 | 40 | Ap | 209115 | H | 53 | 42 | 13 | 1.6 | NA | NA | NA | NA | NA | NA | NA | NA | NA | NA | NA | NA | NA | NA |
| Río Jaltepec | 17.38844 | -95.05611 | 40 | Ap | 209116 | M | 45 | 38 | 11 | 1.4 | NA | NA | NA | NA | NA | NA | NA | NA | NA | NA | NA | NA | NA | NA |
| Río Jaltepec | 17.38844 | -95.05611 | 40 | Ap | 209117 | H | 45 | 35 | 15 | 1.4 | NA | NA | NA | NA | NA | NA | NA | NA | NA | NA | 5 | NA | NA | NA |
| Río Jaltepec | 17.38844 | -95.05611 | 40 | Ap | 209125 | H | 52 | 43 | 10 | 1 | NA | NA | 1 | NA | NA | NA | NA | NA | NA | NA | NA | NA | NA | NA |
| Río Jaltepec | 17.38844 | -95.05611 | 40 | Ap | 209126 | M | 50 | 42 | 10 | 1 | NA | NA | NA | NA | NA | NA | NA | NA | NA | NA | NA | NA | NA | NA |
| Río Jaltepec | 17.38844 | -95.05611 | 40 | Ap | 209120 | M | 50 | 42 | 12 | 1.6 | NA | NA | 1 | NA | 2 | NA | NA | NA | NA | NA | NA | NA | NA | NA |
| Río Jaltepec | 17.38844 | -95.05611 | 40 | Ap | 209121 | H | 43 | 38 | 9 | 1.1 | NA | NA | NA | NA | NA | NA | NA | NA | NA | NA | NA | NA | NA | NA |
| Río Jaltepec | 17.38844 | -95.05611 | 40 | Ap | 209122 | H | 42 | 38 | 9 | 1.5 | NA | NA | 1 | NA | NA | NA | NA | NA | NA | NA | NA | NA | NA | NA |
| Río Jaltepec | 17.38844 | -95.05611 | 40 | Ap | 209123 | H | 40 | 30 | 10 | 1 | NA | NA | NA | NA | NA | NA | NA | NA | NA | NA | NA | NA | NA | NA |
| Río Pánfilo | 17.08364 | -94.87394 | 60 | Ap | 20901 | M | 105 | 85 | 30 | NA | NA | NA | NA | NA | NA | NA | NA | NA | NA | NA | NA | NA | NA | NA |
| Río Pánfilo | 17.08364 | -94.87394 | 60 | Ap | 20902 | M | 90 | 70 | 35 | 6.2 | NA | NA | NA | NA | NA | NA | NA | NA | NA | NA | NA | NA | NA | NA |
| Río Pánfilo | 17.08364 | -94.87394 | 60 | Ap | 20903 | F | 95 | 78 | 27 | 11.6 | NA | NA | NA | NA | NA | NA | NA | NA | NA | NA | NA | NA | 2 | NA |
| Río Pánfilo | 17.08364 | -94.87394 | 60 | Ap | 20904 | M | 93 | 78 | 29 | 12.5 | NA | NA | NA | NA | NA | NA | NA | NA | NA | NA | NA | NA | 1 | NA |
| Río Pánfilo | 17.08364 | -94.87394 | 60 | Ap | 20905 | M | 100 | 80 | 40 | 14.6 | NA | NA | NA | NA | NA | NA | NA | NA | NA | NA | NA | NA | NA | NA |
| Río Pánfilo | 17.08364 | -94.87394 | 60 | Ap | 20906 | F | 84 | 67 | 23 | 7.5 | 1 | NA | NA | 3 | NA | NA | NA | NA | NA | 4 | NA | NA | NA | NA |
| Río Pánfilo | 17.08364 | -94.87394 | 60 | Ap | 20907 | M | 62 | 50 | 18 | 3.2 | NA | NA | NA | NA | NA | NA | NA | NA | NA | NA | NA | NA | NA | NA |
| Río Pánfilo | 17.08364 | -94.87394 | 60 | Ap | 20908 | M | 75 | 65 | 25 | 5.4 | NA | NA | NA | NA | NA | NA | NA | NA | NA | NA | NA | NA | 3 | NA |
| Río Pánfilo | 17.08364 | -94.87394 | 60 | Ap | 20909 | M | 65 | 57 | 18 | 3.1 | NA | NA | NA | 4 | 1 | NA | NA | NA | NA | NA | NA | NA | NA | NA |
| Río Pánfilo | 17.08364 | -94.87394 | 60 | Ap | 20910 | M | 85 | 60 | 25 | 7.2 | NA | NA | NA | NA | NA | NA | NA | NA | NA | NA | NA | NA | NA | NA |
| Río Pánfilo | 17.08364 | -94.87394 | 60 | Ap | 20911 | F | 63 | 52 | 18 | 3.1 | NA | NA | NA | NA | NA | NA | NA | NA | NA | NA | NA | NA | NA | NA |
| Río Pánfilo | 17.08364 | -94.87394 | 60 | Ap | 20912 | M | 70 | 50 | 20 | 4.4 | NA | NA | NA | NA | NA | NA | NA | NA | NA | NA | NA | NA | NA | NA |
| Río Pánfilo | 17.08364 | -94.87394 | 60 | Ap | 20914 | M | 60 | 52 | 20 | 3.4 | NA | NA | NA | NA | NA | NA | NA | NA | NA | NA | NA | NA | 3 | NA |
| Río Pánfilo | 17.08364 | -94.87394 | 60 | Ap | 20915 | M | 90 | 71 | 36 | 9.9 | NA | NA | 1 | NA | NA | NA | NA | NA | NA | NA | NA | NA | 1 | NA |
| Río Pánfilo | 17.08364 | -94.87394 | 60 | Ap | 20916 | F | 120 | 85 | 40 | 21.4 | NA | NA | NA | NA | NA | NA | NA | NA | NA | NA | NA | NA | NA | NA |
| Río Pánfilo | 17.08364 | -94.87394 | 60 | Ap | 20919 | F | 105 | 80 | 30 | 10.4 | NA | NA | NA | NA | NA | NA | NA | NA | NA | NA | NA | NA | NA | NA |
| Río Pánfilo | 17.08364 | -94.87394 | 60 | Ap | 20920 | M | 70 | 65 | 25 | 4.2 | NA | NA | NA | NA | NA | NA | NA | NA | NA | NA | NA | NA | NA | NA |
| Río Pánfilo | 17.08364 | -94.87394 | 60 | Ap | 20922 | F | 75 | 62 | 27 | 4.4 | NA | NA | NA | NA | NA | NA | NA | NA | NA | NA | NA | NA | NA | NA |
| Río Pánfilo | 17.08364 | -94.87394 | 60 | Ap | 20923 | M | 80 | 65 | 19 | 7.5 | NA | NA | NA | NA | NA | NA | NA | NA | NA | NA | NA | NA | NA | NA |
| Río Pánfilo | 17.08364 | -94.87394 | 60 | Ap | 20923' | M | 90 | 65 | 25 | 3.8 | NA | NA | NA | NA | NA | NA | NA | NA | NA | NA | NA | NA | NA | NA |

**CICHLIDAE**

| **Table_4B_*Cichlosoma_salvini*** |  |  |  |  |  |  |  |  |  |  |  |  |  |  |  |  |  |
| --- | --- | --- | --- | --- | --- | --- | --- | --- | --- | --- | --- | --- | --- | --- | --- | --- | --- |
| Locality | Latitude | Longitude | Alt | Date | Host # | Sex | Tl | Sl | Md | We | *Cci* | *Ctr* | *Dip* | *Pmi* | *Tyl* | *Rkr* | Acu |
| Río Modelo | 17.13478 | -94.745 | 115 | Ma | 109237 | M | 103 | 90 | 36 | 27 | 6 | NA | NA | NA | NA | NA | NA |
| Rio Grande | 16.79217 | -95.016083 | 451 | Ma | 10973 | M | 145 | 120 | 50 | 44.3 | NA | 80 | NA | NA | NA | NA | 1 |
| Rio Negro | 16.89853 | -94.693694 | 421 | Ma | 109167 | F | 67 | 50 | 22 | 4.9 | NA | NA | NA | NA | NA | NA | NA |
| Rio Negro | 16.89853 | -94.693694 | 421 | Ma | 109166 | M | 100 | 80 | 33 | 14.5 | NA | NA | NA | NA | NA | NA | NA |
| Rio Negro | 16.89853 | -94.693694 | 421 | Ma | 109155 | F | 130 | 105 | 55 | 30.8 | NA | NA | 1 | NA | NA | NA | NA |
| Rio Negro | 16.89853 | -94.693694 | 421 | Ma | 109164 | M | 81 | 65 | 31 | 9.5 | NA | NA | NA | NA | NA | NA | NA |
| Rio Negro | 16.89853 | -94.693694 | 421 | Ma | 109157 | M | 118 | 97 | 39 | 23.5 | NA | NA | NA | NA | NA | NA | NA |
| Rio Negro | 16.89853 | -94.693694 | 421 | Ma | 109163 | NA | 112 | 88 | 36 | 19.8 | NA | NA | NA | NA | NA | NA | NA |
| Rio Negro | 16.89853 | -94.693694 | 421 | Ma | 109158 | F | 116 | 96 | 43 | 28.2 | NA | NA | NA | NA | NA | NA | NA |
| Rio Negro | 16.89853 | -94.693694 | 421 | Ma | 109153 | M | 99 | 74 | 34 | 14.1 | NA | NA | NA | NA | NA | NA | NA |
| Rio Negro | 16.89853 | -94.693694 | 421 | Ma | 109149 | F | 58 | 43 | 23 | 3.3 | NA | NA | NA | NA | NA | NA | NA |
| Río Jaltepec | 17.38844 | -95.056111 | 40 | Ap | 209118 | F | 78 | 60 | 24 | 7.5 | NA | NA | 17 | 23 | NA | NA | NA |
| Río Jaltepec | 17.38844 | -95.056111 | 40 | Ap | 209119 | F | 120 | 94 | 38 | 25.5 | 3 | NA | 44 | 2 | 5 | NA | NA |
| Río Jaltepec | 17.38844 | -95.056111 | 40 | Ap | 209124 | M | 70 | 55 | 25 | 7.8 | NA | NA | 8 | NA | NA | 1 | NA |
| Río Jaltepec | 17.38844 | -95.056111 | 40 | Ap | 209127 | M | 75 | 60 | 30 | 9.5 | NA | NA | 22 | NA | NA | NA | NA |
| Río Jaltepec | 17.38844 | -95.056111 | 40 | Ap | 209130 | F | 85 | 77 | 38 | 8.5 | NA | NA | * | * | NA | NA | NA |
| Río Jaltepec | 17.38844 | -95.056111 | 40 | Ap | 209131 | J | 55 | 45 | 20 | 3.1 | NA | NA | 16 | NA | NA | NA | NA |
| Río Jaltepec | 17.38844 | -95.056111 | 40 | Ap | 209133 | NA | 60 | 40 | 20 | 2.8 | NA | NA | 11 | NA | NA | NA | NA |
| Río Jaltepec | 17.38844 | -95.056111 | 40 | Ap | 209134 | M | 68 | 56 | 23 | 4.9 | NA | NA | 22 | NA | NA | NA | NA |
| Río Jaltepec | 17.38844 | -95.056111 | 40 | Ap | 209136 | M | 50 | 40 | 16 | 2.3 | NA | NA | NA | NA | NA | NA | NA |
| Río Escondido | 17.09108 | -94.751694 | 83 | Ap | 20968 | M | 105 | 80 | 35 | 19.3 | 6 | NA | NA | NA | NA | NA | NA |
| Río Escondido | 17.09108 | -94.751694 | 83 | Ap | 202969 | F | 125 | 100 | 40 | 30 | NA | NA | NA | NA | NA | NA | NA |
| Río Escondido | 17.09108 | -94.751694 | 83 | Ap | 22970 | NA | 90 | 75 | 35 | 14 | NA | NA | NA | NA | NA | NA | NA |
| Río Pánfilo | 17.08364 | -94.873944 | 60 | Ap | 20941 | M | 70 | 50 | 15 | 2.3 | NA | NA | NA | NA | NA | NA | NA |
| Río Pánfilo | 17.08364 | -94.873944 | 60 | Ap | 20942 | M | 70 | 50 | 15 | 2.7 | NA | NA | NA | NA | NA | NA | NA |
| Río Pánfilo | 17.08364 | -94.873944 | 60 | Ap | 20943 | NA | 90 | 65 | 30 | 14.3 | 6 | NA | NA | NA | NA | NA | NA |

| **Table_4C_*Paraneetroplus_bulleri*** | |  |  |  |  |  |  |  |  |  |  |  |  |  |  |  |  |  |  |  |
| --- | --- | --- | --- | --- | --- | --- | --- | --- | --- | --- | --- | --- | --- | --- | --- | --- | --- | --- | --- | --- |
| Locality | Latitude | Longitude | Alt | Date | Host # | Sex | Tl | Sl | Md | We | *Sci* | *Cci* | *Pmi* | *Uam* | *Cuc* | Phi | *Rkr* | *Rki* | Acu | *Con* |
| El Platanillo | 16.95111 | -95.24417 | 346 | Ma | 10901 | F | 100 | 82 | 27 | 13.7 | NA | NA | NA | 12 | NA | NA | NA | NA | 2 | NA |
| El Platanillo | 16.95111 | -95.24417 | 346 | Ma | 10938 | F | 90 | 76 | 27 | 9.4 | NA | NA | NA | NA | NA | NA | NA | NA | NA | NA |
| Río Escondido | 17.09108 | -94.75169 | 83 | Ap | 20940 | M | 120 | 95 | 43 | 33.7 | 5 | 2 | NA | NA | NA | NA | 3 | 1 | NA | NA |
| Río Escondido | 17.09108 | -94.75169 | 83 | Ap | 20984 | M | 100 | 75 | 35 | 18.3 | NA | NA | NA | NA | NA | NA | NA | NA | NA | NA |
| Río Escondido | 17.09108 | -94.75169 | 83 | Ap | 20985 | M | 110 | 80 | 40 | 23 | NA | NA | NA | NA | NA | NA | NA | NA | NA | NA |
| Río Escondido | 17.09108 | -94.75169 | 83 | Ap | 20986 | F | 99 | 81 | 35 | 23.6 | NA | NA | NA | NA | NA | NA | 8 | NA | NA | NA |
| Río Escondido | 17.09108 | -94.75169 | 83 | Ap | 20987 | NA | 102 | 82 | 39 | 24.6 | 5 | NA | NA | NA | NA | 1 | 6 | NA | NA | NA |
| Río Escondido | 17.09108 | -94.75169 | 83 | Ap | 20988 | J | 95 | 65 | 40 | 16.6 | NA | NA | NA | NA | NA | NA | NA | NA | NA | NA |
| Río Escondido | 17.09108 | -94.75169 | 83 | Ap | 20989 | M | 94 | 76 | 33 | 15.3 | NA | NA | NA | NA | NA | NA | NA | 1 | NA | NA |
| Río Escondido | 17.09108 | -94.75169 | 83 | Ap | 20990 | M | 162 | 136 | 58 | 79.3 | NA | NA | NA | NA | NA | NA | 8 | 1 | NA | 1 |
| Río Escondido | 17.09108 | -94.75169 | 83 | Ap | 20991 | M | 100 | 87 | 35 | 15.7 | NA | NA | 1 | NA | NA | NA | NA | 9 | NA | 1 |
| Río Escondido | 17.09108 | -94.75169 | 83 | Ap | 20992 | F | 81 | 66 | 29 | 12.5 | NA | 11 | 1 | NA | NA | NA | NA | NA | NA | NA |
| Río Escondido | 17.09108 | -94.75169 | 83 | Ap | 20993 | F | 125 | 95 | 50 | 39.5 | NA | NA | NA | NA | NA | NA | NA | NA | NA | NA |
| Río Escondido | 17.09108 | -94.75169 | 83 | Ap | 20994 | J | 104 | 88 | 38 | 18.3 | NA | NA | NA | NA | NA | NA | NA | 4 | NA | NA |
| Río Escondido | 17.09108 | -94.75169 | 83 | Ap | 20995 | J | 104 | 80 | 39 | 20.3 | 1 | 12 | NA | NA | NA | 1 | 14 | NA | NA | NA |
| Río Escondido | 17.09108 | -94.75169 | 83 | Ap | 20996 | F | 85 | 72 | 31 | 14 | NA | NA | NA | NA | 1 | NA | 17 | 2 | NA | NA |
| Río Escondido | 17.09108 | -94.75169 | 83 | Ap | 20997 | J | 90 | 65 | 35 | 12.6 | NA | NA | NA | NA | NA | NA | 28 | NA | NA | NA |
| Río Escondido | 17.09108 | -94.75169 | 83 | Ap | 20998 | M | 90 | 75 | 34 | 13 | NA | NA | NA | NA | NA | 7 | NA | NA | NA | NA |

| **Table_4D_*Parachromis_friedrischtalii*** | | |  |  |  |  |  |  |  |  |  |  |  |
| --- | --- | --- | --- | --- | --- | --- | --- | --- | --- | --- | --- | --- | --- |
| Locality | Latitude | Longitude | Alt | Date | Host # | Sex | Tl | Sl | Md | We | *Cci* | *Pmi* | *Tyl* |
| Río Jaltepec | 17.38844 | -95.05611 | 40 | Ap | 209138 | F | 81 | 64 | 26 | 10.4 | 1 | 3 | 8 |

| **Table_4E_*Thorichthys_callolepis*** | |  |  |  |  |  |  |  |  |  |  |  |  |  |  |  |  |  |
| --- | --- | --- | --- | --- | --- | --- | --- | --- | --- | --- | --- | --- | --- | --- | --- | --- | --- | --- |
| Locality | Latitude | Longitude | Alt | Date | Host # | Sex | Tl | Sl | Md | We | *Gyr* | *Sci* | *Cfo* | *Cli* | *Dip* | *Cic* | *Con* | *Spi* |
| Rio Negro | 16.89853 | -94.693694 | 421 | Ma | 109116 | M | 130 | 110 | 40 | 27.4 | NA | NA | NA | 5 | 63 | NA | NA | NA |
| Rio Negro | 16.89853 | -94.693694 | 421 | Ma | 109120 | F | 115 | 95 | 40 | 20.5 | NA | NA | NA | NA | 2 | 1 | NA | NA |
| Rio Negro | 16.89853 | -94.693694 | 421 | Ma | 109130 | J | 100 | 80 | 35 | 14.4 | NA | NA | NA | NA | NA | NA | NA | NA |
| Rio Negro | 16.89853 | -94.693694 | 421 | Ma | 109138 | M | 85 | 65 | 25 | 7.7 | NA | NA | NA | NA | NA | NA | NA | NA |
| Rio Negro | 16.89853 | -94.693694 | 421 | Ma | 109123 | M | 110 | 90 | 37 | 18.6 | NA | NA | NA | NA | NA | NA | NA | NA |
| Rio Negro | 16.89853 | -94.693694 | 421 | Ma | 109136 | F | 82 | 55 | 30 | 8.6 | 2 | 1 | NA | NA | NA | NA | NA | NA |
| Rio Negro | 16.89853 | -94.693694 | 421 | Ma | 109128 | M | 85 | 65 | 27 | 8.3 | NA | NA | NA | NA | NA | NA | NA | NA |
| Rio Negro | 16.89853 | -94.693694 | 421 | Ma | 109144 | F | 106 | 74 | 44 | 15.6 | NA | NA | NA | NA | NA | NA | NA | NA |
| Rio Negro | 16.89853 | -94.693694 | 421 | Ma | 109119 | F | 98 | 79 | 34 | 13.5 | NA | NA | NA | NA | 52 | NA | NA | NA |
| Rio Negro | 16.89853 | -94.693694 | 421 | Ma | 109101 | M | 95 | 81 | 31 | 14.4 | NA | NA | NA | NA | NA | NA | NA | NA |
| Rio Negro | 16.89853 | -94.693694 | 421 | Ma | 109146 | F | 76 | 60 | 30 | 9.3 | NA | NA | NA | NA | NA | NA | NA | NA |
| Rio Negro | 16.89853 | -94.693694 | 421 | Ma | 109141 | M | 102 | 78 | 28 | 12.4 | 1 | 1 | 1 | NA | NA | 2 | NA | NA |
| Rio Negro | 16.89853 | -94.693694 | 421 | Ma | 109133 | M | 110 | 93 | 28 | 19.6 | NA | NA | 2 | NA | NA | NA | NA | NA |
| Rio Negro | 16.89853 | -94.693694 | 421 | Ma | 109121 | M | 101 | 84 | 32 | 15.5 | NA | NA | 2 | NA | 2 | NA | NA | NA |
| Rio Negro | 16.89853 | -94.693694 | 421 | Ma | 109143 | M | 83 | 68 | 29 | 9.5 | NA | 4 | NA | NA | NA | NA | NA | NA |
| Rio Negro | 16.89853 | -94.693694 | 421 | Ma | 109135 | M | 98 | 81 | 33 | 15.3 | NA | NA | NA | NA | NA | NA | NA | NA |
| Rio Negro | 16.89853 | -94.693694 | 421 | Ma | 109131 | F | 126 | 99 | 39 | 27.9 | NA | NA | NA | NA | NA | NA | NA | NA |
| Rio Negro | 16.89853 | -94.693694 | 421 | Ma | 109126 | J | 103 | 88 | 28 | 11.9 | NA | NA | NA | 1 | NA | NA | NA | NA |
| Rio Negro | 16.89853 | -94.693694 | 421 | Ma | 109127 | M | 105 | 85 | 32 | 14.4 | NA | NA | NA | NA | 1 | NA | NA | NA |
| Rio Negro | 16.89853 | -94.693694 | 421 | Ma | 109124 | F | 120 | 100 | 50 | 22.9 | NA | NA | NA | NA | NA | NA | NA | NA |
| Rio Negro | 16.89853 | -94.693694 | 421 | Ma | 109133 | M | 90 | 75 | 25 | 3.4 | NA | 1 | NA | NA | NA | NA | NA | NA |
| Rio Negro | 16.89853 | -94.693694 | 421 | Ma | 109137 | M | 115 | 93 | 38 | 23 | NA | NA | NA | NA | NA | NA | NA | 1 |
| Rio Negro | 16.89853 | -94.693694 | 421 | Ma | 109134 | NA | 90 | 75 | 30 | 10 | NA | NA | NA | NA | NA | 3 | NA | NA |
| Rio Negro | 16.89853 | -94.693694 | 421 | Ma | 109132 | M | 100 | 80 | 30 | 13 | NA | NA | NA | NA | 2 | NA | NA | NA |
| Rio Negro | 16.89853 | -94.693694 | 421 | Ma | 109129 | F | 80 | 66 | 26 | 8.1 | NA | NA | NA | 1 | NA | NA | NA | NA |
| Rio Negro | 16.89853 | -94.693694 | 421 | Ma | 109125 | M | 73 | 61 | 25 | 8.3 | NA | NA | 2 | NA | NA | 4 | NA | NA |
| Rio Negro | 16.89853 | -94.693694 | 421 | Ma | 10996 | M | 30 | 22 | 11 | 7.5 | NA | NA | NA | NA | NA | 4 | NA | NA |
| Rio Negro | 16.89853 | -94.693694 | 421 | Ma | 109140 | M | 96 | 70 | 30 | 10 | NA | NA | NA | NA | 2 | 1 | NA | NA |
| Rio Negro | 16.89853 | -94.693694 | 421 | Ma | 109109 | M | 97 | 71 | 38 | 13.3 | NA | NA | NA | NA | NA | NA | NA | NA |
| Rio Negro | 16.89853 | -94.693694 | 421 | Ma | 109102 | M | 103 | 85 | 36 | 19.3 | NA | NA | NA | NA | NA | NA | NA | NA |
| Río Jaltepec | 17.38844 | -95.056111 | 40 | Ap | 209140 | J | 60 | 49 | 19 | 3.4 | NA | NA | NA | NA | 2 | NA | 1 | NA |
| Río Jaltepec | 17.38844 | -95.056111 | 40 | Ap | 209148 | NA | 50 | 38 | 12 | 1.6 | NA | NA | NA | NA | 3 | NA | NA | NA |
| Río Jaltepec | 17.38844 | -95.056111 | 40 | Ap | 209150 | M | 58 | 45 | 21 | 1.9 | NA | NA | NA | NA | 7 | NA | NA | NA |
| Río Jaltepec | 17.38844 | -95.056111 | 40 | Ap | 209151 | NA | 50 | 40 | 18 | 2 | NA | NA | NA | NA | 3 | NA | 2 | NA |
| Río Jaltepec | 17.38844 | -95.056111 | 40 | Ap | 209152 | J | 42 | 33 | 13 | 1.2 | NA | NA | NA | NA | NA | NA | NA | NA |
| Río Jaltepec | 17.38844 | -95.056111 | 40 | Ap | 209153 | J | 50 | 35 | 20 | 2.2 | NA | NA | NA | NA | NA | NA | NA | NA |
| Río Jaltepec | 17.38844 | -95.056111 | 40 | Ap | 209154 | NA | 40 | 30 | 15 | 1.2 | NA | NA | NA | NA | 3 | NA | NA | NA |

| **Table_4F_*Theraphs irregularis*** | | |  |  |  |  |  |  |  |  |  |  |
| --- | --- | --- | --- | --- | --- | --- | --- | --- | --- | --- | --- | --- |
| Locality | Latitude | Longitude | Alt | Date | Host # | Sex | Tl | Sl | Md | We | Phi | *Rha* |
| Rio Negro | 16.89853 | -94.69369 | 421 | Ma | 1019173 | M | 136 | 113 | 38 | 37.8 | 1 | 7 |
| Rio Negro | 16.89853 | -94.69369 | 421 | Ma | 109175 | F | 125 | 100 | 50 | 28.4 | NA | NA |
| Rio Negro | 16.89853 | -94.69369 | 421 | Ma | 109170 | M | 155 | 125 | 45 | 42 | NA | 3 |
| Rio Negro | 16.89853 | -94.69369 | 421 | Ma | 109177 | F | 73 | 65 | 26 | 6.2 | NA | NA |
| Rio Negro | 16.89853 | -94.69369 | 421 | Ma | 109176 | M | 154 | 126 | 46 | 45 | NA | NA |
| Rio Negro | 16.89853 | -94.69369 | 421 | Ma | 109168 | F | 150 | 120 | 50 | 53.3 | NA | 8 |

| **Table_4G_*Thorichthys_ellioti*** | |  |  |  |  |  |  |  |  |  |  |
| --- | --- | --- | --- | --- | --- | --- | --- | --- | --- | --- | --- |
| Locality | Latitude | Longitude | Alt | Date | Host # | Sex | Tl | Sl | Md | We | *Pre* |
| Río Escondido | 17.09108 | -94.75169 | 83 | Ap | 20970 | NA | 90 | 75 | 35 | 14 | 3 |

| **Table_4H_*Thorichthys_helleri*** | |  |  |  |  |  |  |  |  |  |  |  |  |  |  |  |  |  |
| --- | --- | --- | --- | --- | --- | --- | --- | --- | --- | --- | --- | --- | --- | --- | --- | --- | --- | --- |
| Locality | Latitude | Longitude | Alt | Date | Host # | Sex | Tl | Sl | Md | We | *Cci* | *Gis* | *Ctr* | *Cuc* | *Pre* | *Rkr* | *Con* | *Rha* |
| Río Modelo | 17.13478 | -94.745 | 115 | Ma | 109236 | F | 81 | 62 | 27 | 8 | NA | NA | NA | NA | NA | NA | 1 | NA |
| Río Modelo | 17.13478 | -94.745 | 115 | Ma | 109240 | M | 68 | 54 | 24 | 4.9 | NA | NA | NA | NA | 4 | NA | NA | NA |
| Río Modelo | 17.13478 | -94.745 | 115 | Ma | 109226 | M | 200 | 163 | 74 | 181 | NA | NA | 2 | 5 | NA | hundreds | NA | 3 |
| Río Modelo | 17.13478 | -94.745 | 115 | Ma | 109232 | J | 100 | 80 | 40 | 12.3 | 1 | NA | NA | NA | NA | NA | NA | 1 |
| Río Modelo | 17.13478 | -94.745 | 115 | Ma | 109229 | F | 107 | 74 | 36 | 15.3 | NA | NA | NA | NA | 3 | NA | NA | NA |
| Río Modelo | 17.13478 | -94.745 | 115 | Ma | 109228 | M | 228 | 195 | 98 | 211.5 | NA | NA | 3 | NA | NA | Hundreds | NA | NA |
| Río Modelo | 17.13478 | -94.745 | 115 | Ma | 109233 | NA | 119 | 88 | 45 | 24.9 | 1 | NA | NA | NA | NA | NA | NA | NA |
| Río Modelo | 17.13478 | -94.745 | 115 | Ma | 109231 | J | 88 | 67 | 29 | 10.6 | 2 | 5 | NA | NA | 1 | NA | 1 | NA |
| Rio Grande | 16.79217 | -95.016083 | 451 | Ma | 10976 | M | 90 | 70 | 30 | 12.1 | NA | NA | NA | NA | 1 | NA | NA | NA |
| Rio Grande | 16.79217 | -95.016083 | 451 | Ma | 10982 | M | 90 | 70 | 27 | 10.4 | NA | NA | NA | NA | 3 | NA | NA | NA |
| Rio Grande | 16.79217 | -95.016083 | 451 | Ma | 10985 | M | 62 | 42 | 20 | 3.4 | NA | NA | NA | NA | NA | NA | NA | NA |

| **Table_4I_*Vieja_guttulata*** | |  |  |  |  |  |  |  |  |  |  |  |  |  |  |  |  |  |  |  |  |  |  |  |  |
| --- | --- | --- | --- | --- | --- | --- | --- | --- | --- | --- | --- | --- | --- | --- | --- | --- | --- | --- | --- | --- | --- | --- | --- | --- | --- |
| Locality | Latitude | Longitude | Alt | Date | Host # | Sex | Tl | Sl | Md | We | *Sci* | *Gyr* | *Gis* | *Cli* | *Dip* | *Pos* | *Cic* | *Sac* | *Avi* | *Can* | *Cuc* | *Rkr* | *Rki* | *Hce* | *Con* |
| Rio Negro | 16.89853 | -94.693694 | 421 | Ma | 109195 | M | 100 | 79 | 32 | 14 | NA | NA | NA | NA | NA | NA | 1 | NA | NA | NA | NA | NA | NA | NA | NA |
| Rio Negro | 16.89853 | -94.693694 | 421 | Ma | 109181 | F | 112 | 92 | 38 | 21.2 | NA | NA | NA | NA | NA | NA | NA | NA | NA | NA | NA | NA | NA | NA | NA |
| Rio Negro | 16.89853 | -94.693694 | 421 | Ma | 109178 | F | 98 | 79 | 38 | 17 | NA | NA | NA | NA | NA | NA | NA | NA | NA | NA | NA | NA | NA | NA | NA |
| Rio Negro | 16.89853 | -94.693694 | 421 | Ma | 109184 | F | 164 | 128 | 59 | 83.4 | NA | NA | NA | NA | NA | NA | NA | NA | NA | 2 | NA | NA | NA | NA | NA |
| Rio Negro | 16.89853 | -94.693694 | 421 | Ma | 109190 | NA | 116 | 96 | 46 | 34.2 | NA | NA | NA | NA | NA | NA | NA | NA | NA | NA | NA | NA | NA | NA | NA |
| Rio Negro | 16.89853 | -94.693694 | 421 | Ma | 109198 | NA | 105 | 82 | 37 | 16.9 | NA | NA | NA | NA | NA | NA | NA | NA | NA | NA | NA | NA | NA | NA | NA |
| Rio Negro | 16.89853 | -94.693694 | 421 | Ma | 109204 | F | 153 | 118 | 58 | 64.2 | NA | NA | NA | NA | NA | NA | NA | NA | NA | 1 | NA | 27 | NA | NA | NA |
| Rio Negro | 16.89853 | -94.693694 | 421 | Ma | 109206 | M | 154 | 120 | 50 | 65.2 | NA | NA | NA | NA | NA | NA | NA | NA | NA | 4 | NA | NA | NA | NA | NA |
| Rio Negro | 16.89853 | -94.693694 | 421 | Ma | 109189 | F | 200 | 160 | 66 | 145.6 | NA | NA | 1 | 1 | 6 | NA | NA | NA | NA | 2 | NA | NA | NA | 2 | NA |
| Rio Negro | 16.89853 | -94.693694 | 421 | Ma | 109211 | J | 95 | 80 | 40 | 12.7 | NA | NA | NA | NA | 2 | NA | NA | NA | NA | NA | NA | NA | NA | NA | NA |
| Rio Negro | 16.89853 | -94.693694 | 421 | Ma | 109202 | M | 120 | 95 | 55 | 27.6 | NA | NA | NA | NA | NA | NA | NA | NA | NA | 1 | NA | NA | 5 | NA | NA |
| Rio Negro | 16.89853 | -94.693694 | 421 | Ma | 109197 | F | 110 | 85 | 45 | 20.7 | NA | NA | NA | NA | NA | NA | NA | NA | NA | NA | NA | NA | NA | NA | NA |
| Rio Negro | 16.89853 | -94.693694 | 421 | Ma | 109193 | J | 85 | 65 | 35 | 68 | NA | NA | NA | NA | NA | NA | NA | NA | NA | NA | NA | NA | NA | NA | NA |
| Rio Negro | 16.89853 | -94.693694 | 421 | Ma | 109183 | J | 95 | 75 | 40 | 12.1 | NA | NA | NA | NA | 1 | NA | NA | NA | NA | NA | NA | NA | NA | NA | NA |
| Rio Negro | 16.89853 | -94.693694 | 421 | Ma | 109205 | M | 93 | 72 | 35 | 16 | NA | NA | NA | NA | NA | NA | NA | NA | NA | NA | NA | 11 | 12 | NA | NA |
| Rio Negro | 16.89853 | -94.693694 | 421 | Ma | 109203 | M | 65 | 48 | 22 | 5.1 | NA | NA | NA | NA | NA | 1 | NA | NA | NA | NA | NA | NA | NA | NA | NA |
| Rio Negro | 16.89853 | -94.693694 | 421 | Ma | 109192 | M | 130 | 104 | 45 | 41 | NA | NA | NA | NA | NA | NA | NA | NA | NA | NA | NA | 3 | 3 | NA | NA |
| Rio Negro | 16.89853 | -94.693694 | 421 | Ma | 109182 | F | 72 | 61 | 27 | 29 | NA | NA | NA | NA | 1 | 3 | NA | NA | NA | NA | NA | 10 | NA | NA | NA |
| Rio Negro | 16.89853 | -94.693694 | 421 | Ma | 109179 | M | 70 | 60 | 26 | 6.4 | NA | NA | NA | NA | NA | NA | NA | NA | NA | NA | NA | NA | NA | NA | NA |
| Rio Negro | 16.89853 | -94.693694 | 421 | Ma | 109186 | F | 73 | 54 | 26 | 7.3 | NA | NA | NA | NA | NA | NA | NA | NA | NA | NA | NA | NA | NA | NA | NA |
| Rio Negro | 16.89853 | -94.693694 | 421 | Ma | 109208 | F | 114 | 87 | 41 | 24.4 | NA | NA | NA | NA | NA | NA | NA | NA | 37 | 2 | NA | 2 | 3 | NA | NA |
| Rio Negro | 16.89853 | -94.693694 | 421 | Ma | 109209 | F | 142 | 123 | 38 | 19.2 | NA | NA | NA | NA | NA | NA | NA | NA | NA | NA | NA | 2 | NA | NA | NA |
| Rio Negro | 16.89853 | -94.693694 | 421 | Ma | 109199 | F | 87 | 69 | 28 | 11.6 | NA | NA | NA | NA | NA | NA | NA | NA | NA | NA | NA | NA | NA | NA | NA |
| Rio Negro | 16.89853 | -94.693694 | 421 | Ma | 109185 | F | NA | NA | NA | 14.5 | NA | NA | NA | NA | NA | NA | NA | NA | NA | NA | NA | NA | NA | 1 | NA |
| Rio Negro | 16.89853 | -94.693694 | 421 | Ma | 109200 | M | 150 | 130 | 52 | 67 | NA | NA | NA | 7 | 4 | NA | NA | NA | NA | NA | NA | NA | 1 | NA | NA |
| Rio Negro | 16.89853 | -94.693694 | 421 | Ma | 109194 | M | 145 | 128 | 50 | 19.2 | NA | NA | NA | NA | NA | NA | NA | NA | NA | NA | NA | 1 | NA | NA | NA |
| Rio Negro | 16.89853 | -94.693694 | 421 | Ma | 109207 | J | 81 | 66 | 27 | 10.3 | NA | NA | NA | NA | NA | 2 | NA | NA | NA | NA | NA | NA | 12 | NA | NA |
| Rio Negro | 16.89853 | -94.693694 | 421 | Ma | 109201 | J | 76 | 62 | 28 | 8.6 | NA | NA | 1 | NA | NA | NA | NA | NA | NA | NA | NA | NA | NA | NA | NA |
| Rio Negro | 16.89853 | -94.693694 | 421 | Ma | 109187 | J | 80 | 69 | 27 | 9.1 | NA | 1 | NA | NA | NA | NA | NA | NA | NA | NA | NA | NA | 1 | NA | NA |
| El Platanillo | 16.95111 | -95.244167 | 346 | Ma | 10936 | J | 135 | 115 | 40 | 39.1 | NA | NA | NA | NA | NA | NA | NA | NA | NA | NA | NA | NA | 1 | NA | NA |
| El Platanillo | 16.95111 | -95.244167 | 346 | Ma | 10937 | M | 283 | 242 | 56 | 108.1 | NA | NA | NA | NA | NA | NA | NA | NA | NA | 10 | NA | NA | NA | NA | NA |
| El Platanillo | 16.95111 | -95.244167 | 346 | Ma | 10939 | F | 160 | 120 | 60 | 60 | NA | NA | 1 | NA | NA | NA | NA | NA | NA | 1 | NA | NA | 4 | NA | NA |
| El Platanillo | 16.95111 | -95.244167 | 346 | Ma | 10941 | F | 80 | 78 | 35 | 17.5 | NA | NA | NA | NA | NA | NA | NA | NA | NA | NA | NA | NA | NA | NA | NA |
| El Platanillo | 16.95111 | -95.244167 | 346 | Ma | 10942 | J | 90 | 75 | 40 | 9.1 | NA | NA | NA | NA | NA | NA | NA | NA | NA | NA | NA | NA | 6 | NA | NA |
| El Platanillo | 16.95111 | -95.244167 | 346 | Ma | 10943 | M | 148 | 115 | 50 | 48.2 | NA | NA | NA | NA | NA | NA | NA | NA | NA | NA | NA | NA | NA | NA | NA |
| El Platanillo | 16.95111 | -95.244167 | 346 | Ma | 10944 | M | 141 | 113 | 50 | 44.5 | NA | NA | NA | NA | NA | NA | NA | NA | NA | NA | NA | NA | NA | NA | NA |
| El Platanillo | 16.95111 | -95.244167 | 346 | Ma | 10945 | M | 210 | 175 | 56 | 133.4 | 1 | NA | NA | NA | NA | NA | NA | NA | NA | 1 | NA | NA | NA | NA | NA |
| El Platanillo | 16.95111 | -95.244167 | 346 | Ma | 10946 | M | 120 | 90 | 30 | 23.9 | NA | NA | NA | NA | NA | NA | NA | NA | NA | NA | NA | NA | NA | NA | NA |
| El Platanillo | 16.95111 | -95.244167 | 346 | Ma | 10947 | J | 85 | 78 | 30 | 10.2 | NA | NA | NA | NA | NA | NA | NA | NA | NA | NA | NA | NA | 1 | NA | NA |
| El Platanillo | 16.95111 | -95.244167 | 346 | Ma | 10948 | M | 155 | 116 | 55 | 43 | NA | NA | NA | NA | NA | NA | NA | NA | NA | 1 | NA | NA | 1 | NA | NA |
| El Platanillo | 16.95111 | -95.244167 | 346 | Ma | 10949 | M | 130 | 103 | 43 | 34.6 | NA | NA | NA | NA | NA | NA | NA | NA | NA | NA | NA | NA | NA | NA | NA |
| El Platanillo | 16.95111 | -95.244167 | 346 | Ma | 10950 | M | 152 | 120 | 50 | 50.5 | NA | NA | NA | NA | NA | NA | NA | NA | NA | NA | NA | NA | 73 | NA | NA |
| El Platanillo | 16.95111 | -95.244167 | 346 | Ma | 10951 | J | 98 | 70 | 40 | 12.3 | NA | NA | NA | NA | NA | NA | NA | NA | NA | NA | NA | NA | NA | NA | NA |
| El Platanillo | 16.95111 | -95.244167 | 346 | Ma | 10952 | F | 71 | 58 | 21 | 7.8 | NA | NA | NA | NA | NA | NA | NA | NA | NA | 2 | NA | NA | NA | NA | NA |
| El Platanillo | 16.95111 | -95.244167 | 346 | Ma | 10953 | J | 83 | 70 | 30 | 9.9 | NA | NA | NA | NA | NA | NA | NA | NA | NA | NA | NA | NA | 1 | NA | NA |
| El Platanillo | 16.95111 | -95.244167 | 346 | Ma | 10954 | M | 80 | 68 | 26 | 9.8 | 1 | NA | NA | NA | NA | NA | NA | NA | NA | 1 | NA | NA | 6 | NA | NA |
| El Platanillo | 16.95111 | -95.244167 | 346 | Ma | 10955 | F | 72 | 59 | 23 | 7.2 | 3 | NA | NA | NA | NA | NA | NA | NA | NA | 1 | NA | NA | 4 | NA | NA |
| El Platanillo | 16.95111 | -95.244167 | 346 | Ma | 10956 | M | 90 | 70 | 30 | 2.7 | NA | NA | NA | NA | NA | NA | NA | NA | NA | NA | NA | NA | NA | NA | NA |
| El Platanillo | 16.95111 | -95.244167 | 346 | Ma | 10957 | F | 87 | 76 | 30 | 12.1 | NA | NA | NA | NA | NA | NA | NA | NA | NA | NA | NA | NA | 3 | NA | NA |
| El Platanillo | 16.95111 | -95.244167 | 346 | Ma | 10958 | F | 150 | 125 | 52 | 53.5 | NA | NA | NA | NA | NA | NA | NA | NA | NA | NA | NA | NA | NA | NA | NA |
| El Platanillo | 16.95111 | -95.244167 | 346 | Ma | 10959 | M | 140 | 115 | 50 | 42.1 | NA | NA | NA | NA | NA | NA | NA | NA | NA | 1 | NA | NA | NA | NA | NA |
| El Platanillo | 16.95111 | -95.244167 | 346 | Ma | 10960 | M | 150 | 125 | 58 | 45.9 | NA | NA | NA | NA | NA | NA | NA | NA | NA | NA | NA | NA | NA | NA | NA |
| El Platanillo | 16.95111 | -95.244167 | 346 | Ma | 10963 | M | 76 | 58 | 24 | 7.4 | 1 | NA | NA | NA | NA | NA | NA | NA | NA | NA | NA | NA | 1 | NA | NA |
| Río Jaltepec | 17.38844 | -95.056111 | 40 | Ap | 209155 | J | 43 | 34 | 13 | 1.5 | NA | NA | NA | NA | NA | NA | NA | NA | NA | NA | NA | NA | NA | NA | NA |
| Río Jaltepec | 17.38844 | -95.056111 | 40 | Ap | 209156 | M | 75 | 50 | 25 | 7.3 | NA | NA | NA | NA | NA | NA | NA | NA | NA | NA | NA | NA | NA | NA | NA |
| Río Jaltepec | 17.38844 | -95.056111 | 40 | Ap | 209157 | J | 49 | 40 | 18 | 2.1 | 1 | NA | NA | NA | NA | NA | NA | NA | NA | NA | NA | NA | NA | NA | NA |
| Río Jaltepec | 17.38844 | -95.056111 | 40 | Ap | 209159 | J | 63 | 51 | 28 | 3.8 | NA | NA | NA | NA | NA | 7 | NA | NA | NA | NA | NA | NA | NA | NA | 1 |
| Río Jaltepec | 17.38844 | -95.056111 | 40 | Ap | 209160 | NA | 66 | 54 | 22 | 4.9 | NA | NA | NA | NA | 1 | NA | NA | 1 | NA | NA | NA | NA | NA | NA | NA |
| Río Jaltepec | 17.38844 | -95.056111 | 40 | Ap | 209161 | J | 45 | 35 | 15 | 1.8 | NA | NA | NA | NA | NA | NA | NA | NA | NA | NA | NA | NA | NA | NA | 1 |
| Río Escondido | 17.09108 | -94.751694 | 83 | Ap | 20945 | M | 90 | 70 | 45 | 17.8 | NA | NA | NA | NA | NA | NA | NA | NA | NA | NA | NA | NA | NA | NA | 1 |
| Río Escondido | 17.09108 | -94.751694 | 83 | Ap | 20947 | F | 105 | 87 | 40 | 22.7 | NA | NA | NA | NA | NA | NA | NA | NA | NA | NA | NA | 13 | NA | NA | NA |
| Río Escondido | 17.09108 | -94.751694 | 83 | Ap | 20948 | M | 110 | 80 | 45 | 25.5 | NA | NA | NA | NA | NA | NA | NA | NA | NA | NA | NA | NA | NA | NA | 1 |
| Río Escondido | 17.09108 | -94.751694 | 83 | Ap | 20950 | F | 98 | 80 | 33 | 20.6 | NA | NA | NA | NA | NA | NA | NA | NA | NA | NA | NA | NA | 1 | NA | 1 |
| Río Escondido | 17.09108 | -94.751694 | 83 | Ap | 20951 | M | 95 | 70 | 45 | 17.7 | NA | NA | NA | NA | NA | NA | NA | NA | NA | NA | NA | NA | NA | NA | NA |
| Río Escondido | 17.09108 | -94.751694 | 83 | Ap | 20952 | F | 100 | 83 | 35 | 20 | NA | NA | NA | NA | NA | NA | NA | NA | NA | NA | NA | NA | 1 | NA | NA |
| Río Escondido | 17.09108 | -94.751694 | 83 | Ap | 20953 | M | 90 | 70 | 45 | 16 | NA | NA | NA | NA | NA | NA | NA | NA | NA | NA | NA | NA | NA | NA | NA |
| Río Escondido | 17.09108 | -94.751694 | 83 | Ap | 20954 | M | 78 | 65 | 25 | 10.3 | NA | NA | NA | NA | NA | NA | NA | NA | NA | NA | 1 | NA | NA | NA | NA |
| Río Escondido | 17.09108 | -94.751694 | 83 | Ap | 20955 | F | 75 | 60 | 23 | 8.8 | NA | NA | NA | NA | NA | NA | NA | NA | NA | NA | NA | NA | NA | NA | NA |
| Río Escondido | 17.09108 | -94.751694 | 83 | Ap | 20983 | F | 75 | 60 | 30 | 8.1 | NA | NA | NA | NA | NA | NA | NA | NA | NA | NA | NA | NA | NA | NA | NA |

| **Table_4J_*Vieja_regani*** |  |  |  |  |  |  |  |  |  |  |  |  |  |  |  |  |
| --- | --- | --- | --- | --- | --- | --- | --- | --- | --- | --- | --- | --- | --- | --- | --- | --- |
| Locality | Latitude | Longitude | Alt | Date | Host # | Sex | Tl | Sl | Md | We | *Cci* | *Gis* | *Pos* | *Rkr* | *Rki* | *Con* |
| Río Modelo | 17.13478 | -94.745 | 115 | Ma | 109224 | F | 89 | 72 | 28 | 11.2 | 3 | NA | NA | NA | 1 | NA |
| Río Modelo | 17.13478 | -94.745 | 115 | Ma | 109221 | F | 115 | 95 | 45 | 23.7 | NA | 5 | NA | NA | NA | NA |
| Río Modelo | 17.13478 | -94.745 | 115 | Ma | 109220 | NA | 95 | 75 | 38 | 14.2 | 2 | 16 | NA | NA | NA | NA |
| Río Modelo | 17.13478 | -94.745 | 115 | Ma | 109223 | F | 90 | 78 | 28 | 9 | 2 | NA | NA | NA | 13 | NA |
| Río Modelo | 17.13478 | -94.745 | 115 | Ma | 109225 | J | 68 | 55 | 23 | 6.5 | 2 | 32 | NA | NA | 4 | NA |
| Rio Grande | 16.79217 | -95.0161 | 451 | Ma | 10974 | M | 178 | 130 | 67 | 91.6 | NA | NA | 1 | Thousands | NA | NA |
| Rio Grande | 16.79217 | -95.0161 | 451 | Ma | 10978 | M | 188 | 142 | 63 | 90.8 | NA | NA | NA | Thousands | NA | NA |
| Rio Grande | 16.79217 | -95.0161 | 451 | Ma | 10979 | F | 120 | 98 | 40 | 12 | NA | NA | NA | hundreds | NA | NA |
| Rio Grande | 16.79217 | -95.0161 | 451 | Ma | 10986 | M | 120 | 90 | 40 | 21.3 | NA | NA | NA | NA | NA | NA |
| Rio Grande | 16.79217 | -95.0161 | 451 | Ma | 10987 | M | 150 | 120 | 47 | 52.3 | NA | NA | NA | NA | NA | 1 |

**ELEOTRIDAE**

| **Table_4K_*Gobiomorus_dormitor*** | |  |  |  |  |  |  |  |  |  |  |  |  |  |  |  |  |  |  |
| --- | --- | --- | --- | --- | --- | --- | --- | --- | --- | --- | --- | --- | --- | --- | --- | --- | --- | --- | --- |
| Locality | Latitude | Longitude | Alt | Date | Host # | Sex | Tl | Sl | Md | We | *Gtr* | *Gis* | *Cfo* | *Pte* | *Cuc* | *Con* | *Fal* | *Rha* | *Spi* |
| Río Modelo | 17.13478 | -94.745 | 115 | Ma | 109227 | M | 330 | 290 | 40 | 300 | 12 | NA | NA | NA | NA | 25 | NA | NA | NA |
| Río Modelo | 17.13478 | -94.745 | 115 | Ma | 109241 | F | 130 | 100 | 14 | 14.7 | NA | NA | NA | NA | NA | NA | NA | 1 | NA |
| Río Modelo | 17.13478 | -94.745 | 115 | Ma | 109242 | NA | 164 | 136 | 25 | 330 | NA | NA | NA | NA | NA | 1 | NA | NA | NA |
| Río Modelo | 17.13478 | -94.745 | 115 | Ma | 109244 | J | 121 | 102 | 13 | 11.4 | NA | 2 | NA | NA | NA | 1 | NA | 5 | NA |
| Rio Negro | 16.89853 | -94.693694 | 421 | Ma | 109214 | M | 175 | 152 | 23 | 36.9 | 9 | NA | 13 | NA | NA | 3 | NA | NA | NA |
| Rio Negro | 16.89853 | -94.693694 | 421 | Ma | 109172 | F | 280 | 238 | 37 | 17.9 | 67 | NA | 1 | NA | NA | 6 | 1 | NA | 1 |
| Rio Negro | 16.89853 | -94.693694 | 421 | Ma | 109216 | J | 136 | 109 | 13 | 16.8 | 18 | NA | NA | 3 | NA | 2 | NA | NA | NA |
| Rio Negro | 16.89853 | -94.693694 | 421 | Ma | 109218 | F | 136 | 111 | 16 | 13.9 | NA | NA | NA | NA | NA | NA | NA | NA | NA |
| Rio Negro | 16.89853 | -94.693694 | 421 | Ma | 109213 | F | 140 | 115 | 20 | 16 | NA | NA | 3 | NA | NA | NA | NA | NA | NA |
| Rio Negro | 16.89853 | -94.693694 | 421 | Ma | 109215 | NA | 125 | 110 | 18 | 12 | NA | NA | 5 | NA | NA | NA | NA | NA | NA |
| Río Jaltepec | 17.38844 | -95.056111 | 40 | Ap | 209137 | F | 130 | 100 | 15 | 17.1 | NA | NA | NA | NA | NA | NA | NA | NA | NA |
| Río Escondido | 17.09108 | -94.751694 | 83 | Ap | 20963 | M | 220 | 190 | 23 | 49.9 | 9 | NA | NA | 1 | 1 | 4 | NA | NA | NA |
| Río Pánfilo | 17.08364 | -94.873944 | 60 | Ap | 20917 | M | 160 | 139 | 22 | 33.9 | NA | NA | NA | NA | NA | NA | NA | NA | NA |
| Río Pánfilo | 17.08364 | -94.873944 | 60 | Ap | 20913 | M | 112 | 94 | 15 | 8.7 | NA | NA | NA | NA | NA | NA | NA | 3 | NA |
| Río Pánfilo | 17.08364 | -94.873944 | 60 | Ap | 20918 | M | 128 | 104 | 8 | 14.2 | NA | NA | NA | NA | NA | NA | NA | NA | NA |
| Río Pánfilo | 17.08364 | -94.873944 | 60 | Ap | 20928 | M | 120 | 101 | 14 | 12.1 | NA | NA | NA | NA | NA | 1 | NA | NA | NA |

**GOBIIDAE**

| **Table_4L_*Awaous_banana*** |  |  |  |  |  |  |  |  |  |  |  |  |
| --- | --- | --- | --- | --- | --- | --- | --- | --- | --- | --- | --- | --- |
| Locality | Latitude | Longitude | Alt | Date | Host # | Sex | Tl | Sl | Md | We | *Nch* | *Con* |
| Río Modelo | 17.13478 | -94.745 | 115 | Ma | 109222 | F | 210 | 176 | 42 | 82.4 | NA | NA |
| Rio Negro | 16.89853 | -94.693694 | 421 | Ma | 109162 | F | 130 | 105 | 14 | 18.1 | 8 | NA |
| Rio Negro | 16.89853 | -94.693694 | 421 | Ma | 109165 | F | 100 | 84 | 13 | 8.1 | NA | NA |
| Rio Negro | 16.89853 | -94.693694 | 421 | Ma | 109169 | M | 178 | 151 | 24 | 48 | 7 | NA |
| Rio Negro | 16.89853 | -94.693694 | 421 | Ma | 109154 | M | 142 | 127 | 25 | 28 | 4 | NA |
| Rio Negro | 16.89853 | -94.693694 | 421 | Ma | 109156 | F | 177 | 145 | 23 | 54.7 | 1 | NA |
| Rio Negro | 16.89853 | -94.693694 | 421 | Ma | 109159 | F | 135 | 125 | 20 | 22.5 | 4 | 1 |
| Rio Negro | 16.89853 | -94.693694 | 421 | Ma | 109161 | J | 150 | 120 | 20 | 27 | 3 | NA |
| Rio Negro | 16.89853 | -94.693694 | 421 | Ma | 109160 | F | NA | NA | NA | NA | 2 | NA |

**HEPTAPTERIDAE**

| **Table_4M_*Rhamdia_guatemalensis*** |  |  |  |  |  |  |  |  |  |  |  |  |  |  |  |
| --- | --- | --- | --- | --- | --- | --- | --- | --- | --- | --- | --- | --- | --- | --- | --- |
| Locality | Latitude | Longitude | Altit | Date | Host # | Sex | Tl | Sl | Md | We | *Atr* | *Cps* | *Cli* | *Cme* | *Con* |
| Rio Negro | 16.89853 | -94.693694 | 421 | Ma | 109150 | NA | 120 | 100 | 30 | 12.3 | NA | NA | 1 | NA | NA |
| Rio Negro | 16.89853 | -94.693694 | 421 | Ma | 109122 | H | 164 | 136 | 24 | 30.4 | NA | NA | NA | NA | NA |
| Rio Negro | 16.89853 | -94.693694 | 421 | Ma | 109142 | M | 250 | 220 | 50 | 110.4 | 3 | NA | NA | NA | NA |
| Rio Negro | 16.89853 | -94.693694 | 421 | Ma | 109139 | M | 130 | 100 | 15 | 15.9 | NA | NA | NA | NA | NA |
| Rio Grande | 16.79217 | -95.016083 | 451 | Ma | 10984 | H | 180 | 145 | 29 | 46 | NA | NA | NA | NA | 1 |
| Río Pánfilo | 17.08364 | -94.873944 | 60 | Ap | 20925 | M | 165 | 125 | 25 | 27.7 | NA | NA | 4 | NA | 1 |
| Río Pánfilo | 17.08364 | -94.873944 | 60 | Ap | 20930 | M | 200 | 155 | 30 | 44.9 | NA | NA | 3 | NA | NA |
| Río Pánfilo | 17.08364 | -94.873944 | 60 | Ap | 20934 | F | 238 | 200 | 35 | 115.4 | NA | NA | 80 | NA | NA |
| Río Pánfilo | 17.08364 | -94.873944 | 60 | Ap | 20937 | F | 260 | 205 | 55 | 113.9 | NA | NA | 17 | NA | 6 |
| Río Pánfilo | 17.08364 | -94.873944 | 60 | Ap | 20946 | M | 110 | 90 | 8 | 10.1 | NA | 1 | NA | 1 | NA |

| **Table_4N_*Rhamdia_laticauda*** | |  |  |  |  |  |  |  |  |  |  |  |  |
| --- | --- | --- | --- | --- | --- | --- | --- | --- | --- | --- | --- | --- | --- |
| Locality | Latitude | Longitude | Alt | Date | Host # | Sex | Tl | Sl | Md | We | *Atr* | *Cli* | *Rki* |
| Río Escondido | 17.09108 | -94.751694 | 83 | Ap | 20959 | M | 78 | 68 | 12 | 4.5 | NA | NA | NA |
| Río Escondido | 17.09108 | -94.751694 | 83 | Ap | 20960 | J | 93 | 80 | 13 | 8.9 | NA | NA | 2 |
| Río Escondido | 17.09108 | -94.751694 | 83 | Ap | 20962 | M | 110 | 95 | 20 | 13.8 | NA | NA | NA |
| Río Escondido | 17.09108 | -94.751694 | 83 | Ap | 20967 | NA | 115 | 100 | 25 | 15.7 | NA | NA | NA |
| Río Escondido | 17.09108 | -94.751694 | 83 | Ap | 20973 | NA | 118 | 98 | 13 | 12.1 | 2 | NA | 4 |
| Río Escondido | 17.09108 | -94.751694 | 83 | Ap | 20974 | M | 250 | 195 | 40 | 88 | NA | 2 | NA |

**MUGILIDAE**

| **Table_3O_*Agonostomus_montícola*** |  |  |  |  |  |  |  |  |  |  |  |  |  |
| --- | --- | --- | --- | --- | --- | --- | --- | --- | --- | --- | --- | --- | --- |
| Locality | Latitude | Longitude | Alt | Date | Host # | Sex | Tl | Sl | Md | We | *Cag* | *Sso* | *Dme* |
| Rio Negro | 16.89853 | -94.693694 | 421 | Ma | 109155 | J | 62 | 53 | 9 | 2 | 5 | NA | NA |
| Río Escondido | 17.09108 | -94.751694 | 83 | Ap | 20944 | M | 110 | 93 | 22 | 16.2 | 6 | NA | NA |
| Río Escondido | 17.09108 | -94.751694 | 83 | Ap | 20949 | F | 115 | 98 | 21 | 17 | 4 | NA | NA |
| Río Pánfilo | 17.08364 | -94.873944 | 60 | Ap | 20927 | M | 110 | 92 | 22 | 13.3 | 2 | NA | 4 |
| Río Pánfilo | 17.08364 | -94.873944 | 60 | Ap | 20929 | NA | 80 | 64 | 18 | 4.9 | 4 | 1 | NA |

**POECILIIDAE**

| **Table_4P_*Pseudoxiphophorus_bimaculatus*** | | |  |  |  |  |  |  |  |  |  |  |  |
| --- | --- | --- | --- | --- | --- | --- | --- | --- | --- | --- | --- | --- | --- |
| Locality | Latitude | Longitude | Alt | Date | Host # | Sex | Tl | Sl | Md | We | *Pfe* | *Cfo* | *Sme* |
| Rio Negro | 16.89853 | -94.69369 | 421 | Ma | 109174 | F | 54 | 45 | 12 | 0.8 | NA | NA | NA |
| El Platanillo | 16.95111 | -95.24417 | 346 | Ma | 10907 | M | 60 | 50 | 12 | 2.6 | NA | NA | NA |
| El Platanillo | 16.95111 | -95.24417 | 346 | Ma | 10964 | M | 80 | 68 | 20 | 6.7 | NA | NA | NA |
| El Platanillo | 16.95111 | -95.24417 | 346 | Ma | 10965 | F | 42 | 38 | 9 | 0.9 | NA | NA | NA |
| El Platanillo | 16.95111 | -95.24417 | 346 | Ma | 10967 | M | 45 | 40 | 10 | 1.2 | NA | NA | NA |
| Río Pánfilo | 17.08364 | -94.87394 | 60 | Ap | 20924 | M | 41 | 32 | 10 | 0.9 | NA | NA | NA |
| Río Pánfilo | 17.08364 | -94.87394 | 60 | Ap | 20926 | F | 45 | 38 | 12 | 1.2 | NA | NA | NA |
| Río Pánfilo | 17.08364 | -94.87394 | 60 | Ap | 20931 | F | 51 | 41 | 13 | 1.8 | NA | NA | NA |
| Río Pánfilo | 17.08364 | -94.87394 | 60 | Ap | 20932 | F | 73 | 63 | 15 | 4.5 | NA | NA | NA |
| Río Pánfilo | 17.08364 | -94.87394 | 60 | Ap | 20933 | F | 60 | 54 | 12 | 2 | NA | NA | NA |
| Río Pánfilo | 17.08364 | -94.87394 | 60 | Ap | 20935 | F | 86 | 77 | 18 | 6 | NA | NA | 2 |
| Río Pánfilo | 17.08364 | -94.87394 | 60 | Ap | 20936 | F | 60 | 50 | 12 | 2.6 | NA | NA | NA |
| Río Pánfilo | 17.08364 | -94.87394 | 60 | Ap | 20938 | NA | 48 | 40 | 10 | 1.1 | 14 | 3 | NA |

| **Table_4Q_*Poeciliopsis_gracilis*** | |  |  |  |  |  |  |  |  |  |  |
| --- | --- | --- | --- | --- | --- | --- | --- | --- | --- | --- | --- |
| Locality | Latitude | Longitude | Alt | Date | Host # | Sex | Tl | Sl | Md | We | *Sso* |
| Rio Negro | 16.89853 | -94.693694 | 421 | Ma | 109171 | M | 82 | 38 | 12 | 1.2 | NA |
| Rio Grande | 16.79217 | -95.016083 | 451 | Ma | 10983 | M | 36 | 30 | 5 | 2 | 1 |
| Rio Grande | 16.79217 | -95.016083 | 451 | Ma | 109889 | M | 56 | 47 | 11 | 9 | NA |
| Rio Grande | 16.79217 | -95.016083 | 451 | Ma | 10981 | M | 59 | 49 | 12 | 2.4 | NA |
| Rio Grande | 16.79217 | -95.016083 | 451 | Ma | 10980 | NA | 60 | 50 | 15 | 2.1 | NA |
| Río Escondido | 17.09108 | -94.751694 | 83 | Ap | 20982 | NA | 56 | 47 | 10 | 1 | NA |

| **Table_4R_*Priapella_intermedia*** | |  |  |  |  |  |  |  |  |  |  |
| --- | --- | --- | --- | --- | --- | --- | --- | --- | --- | --- | --- |
| Locality | Latitude | Longitude | Alt | Date | Host # | Host sex | Tl | Sl | Md | We | *Uam* |
| Rio Grande | 16.79217 | -95.01608 | 451 | Ma | 10975 | M | 65 | 55 | 25 | 2.2 | 3 |

| **Table_4S_*Poecilia_mexicana*** | |  |  |  |  |  |  |  |  |  |  |  |  |
| --- | --- | --- | --- | --- | --- | --- | --- | --- | --- | --- | --- | --- | --- |
| Locality | Latitude | Longitude | Alt | Date | Host # | Sex | Tl | Sl | Md | We | *Gyr* | *Fal* | *Spi* |
| Río Escondido | 17.09108 | -94.75169 | 83 | Ap | 20999 | M | 65 | 50 | 15 | 3.4 | NA | NA | NA |
| Río Escondido | 17.09108 | -94.75169 | 83 | Ap | 209100 | M | 63 | 53 | 10 | 3.1 | NA | NA | NA |
| Río Escondido | 17.09108 | -94.75169 | 83 | Ap | 209101 | NA | 80 | 65 | 18 | 6 | NA | NA | NA |
| Río Escondido | 17.09108 | -94.75169 | 83 | Ap | 20102 | M | 58 | 43 | 12 | 2.2 | 2 | NA | NA |
| Río Escondido | 17.09108 | -94.75169 | 83 | Ap | 209103 | M | 70 | 55 | 15 | 4.1 | NA | NA | NA |
| Río Escondido | 17.09108 | -94.75169 | 83 | Ap | 209104 | M | 63 | 50 | 12 | 3.3 | NA | NA | 1 |
| Río Escondido | 17.09108 | -94.75169 | 83 | Ap | 209105 | F | 60 | 50 | 19 | 2.8 | NA | NA | NA |
| Río Escondido | 17.09108 | -94.75169 | 83 | Ap | 209106 | M | 60 | 50 | 15 | 3.1 | NA | NA | NA |
| Río Escondido | 17.09108 | -94.75169 | 83 | Ap | 209107 | M | 62 | 50 | 12 | 2.8 | NA | NA | NA |
| Río Escondido | 17.09108 | -94.75169 | 83 | Ap | 209108 | M | 49 | 38 | 10 | 1.1 | 1 | NA | NA |
| Río Escondido | 17.09108 | -94.75169 | 83 | Ap | 209109 | NA | 50 | 40 | 10 | 1.7 | NA | NA | NA |
| Río Escondido | 17.09108 | -94.75169 | 83 | Ap | 209110 | M | 53 | 46 | 11 | 2.1 | 1 | 1 | NA |
| Río Escondido | 17.09108 | -94.75169 | 83 | Ap | 209111 | M | 55 | 45 | 10 | 1.8 | NA | NA | NA |

| **Table_4T_*Poecilia_shenops*** |  |  |  |  |  |  |  |  |  |  |  |  |  |  |  |
| --- | --- | --- | --- | --- | --- | --- | --- | --- | --- | --- | --- | --- | --- | --- | --- |
| Locality | Latitude | Longitude | Alt | Date | Host # | Sex | Tl | Sl | Md | We | *Sso* | *Adi* | *Dip* | *Pos* | *Glo* |
| Rio Grande | 16.79217 | -95.01608 | 451 | Ma | 109151 | M | 90 | 71 | 23 | 9.5 | NA | NA | NA | NA | NA |
| Rio Grande | 16.79217 | -95.01608 | 451 | Ma | 10981 | M | 59 | 49 | 12 | 2.4 | 1 | 1 | NA | 5 | NA |
| Rio Grande | 16.79217 | -95.01608 | 451 | Ma | 10988 | M | 56 | 47 | 11 | 9 | 1 | NA | NA | NA | NA |
| Rio Grande | 16.79217 | -95.01608 | 451 | Ma | 10980 | M | 60 | 50 | 15 | 2.1 | 1 | NA | NA | NA | NA |
| Rio Grande | 16.79217 | -95.01608 | 451 | Ma | 109151 | M | 90 | 71 | 23 | 9.5 | NA | NA | NA | NA | NA |
| Rio Negro | 16.89853 | -94.69369 | 421 | Ma | 109188 | M | 40 | 34 | 12 | 0.9 | NA | NA | NA | NA | NA |
| Rio Negro | 16.89853 | -94.69369 | 421 | Ma | 109191 | M | 47 | 38 | 10 | 0.5 | NA | NA | NA | NA | NA |
| Rio Negro | 16.89853 | -94.69369 | 421 | Ma | 109152 | M | 87 | 78 | 25 | 10.4 | NA | NA | NA | NA | NA |
| Rio Negro | 16.89853 | -94.69369 | 421 | Ma | 109148 | M | 80 | 70 | 20 | 7 | NA | NA | NA | NA | NA |
| Rio Negro | 16.89853 | -94.69369 | 421 | Ma | 109171 | M | 82 | 38 | 12 | 1.2 | NA | NA | NA | NA | NA |
| Río Modelo | 17.13478 | -94.745 | 115 | Ma | 109238 | M | 72 | 60 | 14 | 3.4 | NA | NA | NA | NA | NA |
| Río Modelo | 17.13478 | -94.745 | 115 | Ma | 109230 | M | 95 | 80 | 25 | 7.8 | NA | NA | NA | NA | NA |
| Río Jaltepec | 17.38844 | -95.05611 | 40 | Ap | 209128 | M | 80 | 69 | 19 | 2.7 | NA | NA | NA | NA | NA |
| Río Jaltepec | 17.38844 | -95.05611 | 40 | Ap | 209129 | M | 60 | 50 | 12 | 2.2 | NA | NA | 8 | NA | NA |
| Río Jaltepec | 17.38844 | -95.05611 | 40 | Ap | 209132 | F | 55 | 46 | 13 | 2.2 | NA | NA | 1 | NA | NA |
| Río Jaltepec | 17.38844 | -95.05611 | 40 | Ap | 209135 | M | 73 | 57 | 16 | 4 | NA | NA | 6 | 1 | NA |
| Río Jaltepec | 17.38844 | -95.05611 | 40 | Ap | 209139 | M | 70 | 58 | 16 | 5.5 | NA | NA | 9 | NA | NA |
| Río Jaltepec | 17.38844 | -95.05611 | 40 | Ap | 209142 | M | 72 | 61 | 18 | 5 | NA | NA | NA | NA | 1 |
| Río Jaltepec | 17.38844 | -95.05611 | 40 | Ap | 209143 | F | 71 | 62 | 17 | 1.6 | NA | NA | 9 | NA | NA |
| Río Jaltepec | 17.38844 | -95.05611 | 40 | Ap | 209144 | M | 50 | 42 | 10 | 1.6 | NA | NA | 5 | NA | NA |
| Río Jaltepec | 17.38844 | -95.05611 | 40 | Ap | 209145 | F | 45 | 39 | 10 | 1.6 | 8 | NA | 1 | NA | NA |
| Río Jaltepec | 17.38844 | -95.05611 | 40 | Ap | 209146 | M | 51 | 42 | 11 | 1.5 | NA | NA | NA | NA | NA |

| **Table_4U_*Xiphophorus_clemenciae*** | |  |  |  |  |  |  |  |  |  |  |  |  |  |
| --- | --- | --- | --- | --- | --- | --- | --- | --- | --- | --- | --- | --- | --- | --- |
| Locality | Latitude | Longitude | Alt | Date | Host # | Sex | Tl | Sl | Md | We | *Sso* | *Uam* | *Cfo* | *Spi* |
| Rio Negro | 16.89853 | -94.693694 | 421 | Ma | 109212 | M | 57 | 46 | 12 | 2 | NA | NA | 2 | NA |
| Rio Negro | 16.89853 | -94.693694 | 421 | Ma | 109196 | F | 49 | 40 | 10 | 0.8 | NA | NA | NA | NA |
| Rio Negro | 16.89853 | -94.693694 | 421 | Ma | 109180 | F | 65 | 52 | 14 | 3.4 | NA | NA | 2 | NA |
| Rio Negro | 16.89853 | -94.693694 | 421 | Ma | 109145 | F | 50 | 42 | 11 | 3 | NA | NA | NA | NA |
| Río Modelo | 17.13478 | -94.745 | 115 | Ma | 109234 | M | 64 | 54 | 15 | 1.5 | NA | NA | NA | NA |
| Río Modelo | 17.13478 | -94.745 | 115 | Ma | 109235 | M | 45 | 38 | 15 | 0.4 | NA | NA | NA | NA |
| Rio Grande | 16.79217 | -95.016083 | 451 | Ma | 10970 | M | 49 | 40 | 11 | 2.1 | NA | 17 | NA | 28 |
| Rio Grande | 16.79217 | -95.016083 | 451 | Ma | 10971 | M | 53 | 44 | 14 | 1.8 | NA | NA | NA | NA |
| Rio Grande | 16.79217 | -95.016083 | 451 | Ma | 10972 | M | 54 | 45 | 15 | 1.6 | 1 | NA | NA | NA |
| Río Escondido | 17.09108 | -94.751694 | 83 | Ap | 20971 | F | 58 | 48 | 12 | 2.7 | NA | NA | NA | NA |
| Río Escondido | 17.09108 | -94.751694 | 83 | Ap | 20972 | F | 59 | 47 | 11 | 2.5 | NA | NA | NA | NA |
| Río Escondido | 17.09108 | -94.751694 | 83 | Ap | 20975 | M | 49 | 46 | 9 | 2 | NA | NA | NA | NA |
| Río Escondido | 17.09108 | -94.751694 | 83 | Ap | 20976 | F | 52 | 47 | 10 | 2.3 | NA | NA | NA | NA |
| Río Escondido | 17.09108 | -94.751694 | 83 | Ap | 20977 | M | 51 | 46 | 9 | 2 | NA | NA | NA | NA |
| Río Escondido | 17.09108 | -94.751694 | 83 | Ap | 20978 | NA | 36 | 29 | 8 | 1 | NA | NA | NA | NA |
| Río Escondido | 17.09108 | -94.751694 | 83 | Ap | 20979 | NA | 58 | 49 | 18 | 1.9 | NA | NA | NA | NA |
| Río Escondido | 17.09108 | -94.751694 | 83 | Ap | 20980 | NA | 36 | 30 | 6 | 1 | NA | NA | NA | NA |
| Río Escondido | 17.09108 | -94.751694 | 83 | Ap | 20981 | NA | 42 | 36 | 8 | 1 | NA | NA | NA | NA |

| **Table_4V_*Xiphophorus_helleri*** | |  |  |  |  |  |  |  |  |  |  |
| --- | --- | --- | --- | --- | --- | --- | --- | --- | --- | --- | --- |
| Locality | Latitude | Longitude | Alt | Date | Host # | Sex | Tl | Sl | Md | We | *Con* |
| Río Jaltepec | 17.38844 | -95.056111 | 40 | Ap | 209147 | M | 65 | 50 | 10 | 2.9 | 1 |
| Río Jaltepec | 17.38844 | -95.056111 | 40 | Ap | 209149 | M | 41 | 33 | 10 | 0.9 | NA |

| **Table_4X_*Xiphophorus_mixei*** | |  |  |  |  |  |  |  |  |  |  |
| --- | --- | --- | --- | --- | --- | --- | --- | --- | --- | --- | --- |
| Locality | Latitude | Longitude | Alt | Date | Host # | Sex | Tl | Sl | Md | We | Acu |
| El Platanillo | 16.95111 | -95.24417 | 346 | Ma | 10915 | M | 67 | 70 | 19 | 5.1 | NA |
| El Platanillo | 16.95111 | -95.24417 | 346 | Ma | 10917 | F | 75 | 63 | 18 | 4 | **1** |
| El Platanillo | 16.95111 | -95.24417 | 346 | Ma | 10921 | M | 60 | 45 | 12 | 1.9 | NA |
| El Platanillo | 16.95111 | -95.24417 | 346 | Ma | 10924 | M | 59 | 49 | 18 | 3.8 | NA |
| El Platanillo | 16.95111 | -95.24417 | 346 | Ma | 10928 | M | 65 | 55 | 20 | 4.5 | NA |

| **Table_4Y_*Xiphophorus_monticolus*** | |  |  |  |  |  |  |  |  |  |  |
| --- | --- | --- | --- | --- | --- | --- | --- | --- | --- | --- | --- |
| Locality | Latitude | Longitude | Alt | Date | Host # | Sex | Tl | Sl | Md | We | *Sal* |
| El Platanillo | 16.95111 | -95.2442 | 346 | Ma | 10925 | M | 57 | 43 | 10 | 1.5 | 20 |
| El Platanillo | 16.95111 | -95.2442 | 346 | Ma | 10927 | M | 58 | 40 | 6 | 1.3 | 3 |
| El Platanillo | 16.95111 | -95.2442 | 346 | Ma | 10929 | H | 63 | 54 | 13 | 3.2 | 27 |
| El Platanillo | 16.95111 | -95.2442 | 346 | Ma | 10930 | M | 65 | 50 | 15 | 2.1 | 7 |
| El Platanillo | 16.95111 | -95.2442 | 346 | Ma | 10932 | H | 48 | 38 | 8 | 1.4 | 1 |
| El Platanillo | 16.95111 | -95.2442 | 346 | Ma | 10933 | H | 60 | 50 | 20 | 4.2 | 1 |
| El Platanillo | 16.95111 | -95.2442 | 346 | Ma | 10934 | H | 53 | 43 | 22 | 1.3 | 1 |
| El Platanillo | 16.95111 | -95.2442 | 346 | Ma | 10935 | M | 61 | 49 | 11 | 2 | 12 |
| El Platanillo | 16.95111 | -95.2442 | 346 | Ma | 10940 | M | 60 | 44 | 12 | NA | NA |

**SYNBRANCHIDAE**

| **Table_4Z_*Ophisternon_aenigmaticum*** | | |  |  |  |  |  |  |  |  |  |  |  |  |
| --- | --- | --- | --- | --- | --- | --- | --- | --- | --- | --- | --- | --- | --- | --- |
| Locality | Latitude | Longitude | Alt | Date | Host # | Sex | Tl | Sl | Md | We | *Pop* | Phi | *Con* | *Rha* |
| Rio Negro | 16.89853 | -94.69369 | 421 | Ma | 109217 | F | 230 | NA | 10 | 13.3 | NA | NA | NA | NA |
| Rio Negro | 16.89853 | -94.69369 | 421 | Ma | 109210 | M | 280 | NA | 17 | 47.7 | NA | 3 | 3 | NA |
| Rio Negro | 16.89853 | -94.69369 | 421 | Ma | 109243 | M | 243 | NA | 22 | 34.5 | NA | NA | NA | NA |
| Río Modelo | 17.13478 | -94.745 | 115 | Ma | 109239 | M | 380 | NA | 29 | 52 | NA | NA | 2 | NA |
| Rio Grande | 16.79217 | -95.01608 | 451 | Ma | 10977 | M | 392 | NA | NA | 68.9 | NA | NA | 1 | NA |
| El Platanillo | 16.95111 | -95.24417 | 346 | Ma | 10961 | M | 255 | 190 | 13 | 20.2 | NA | NA | NA | NA |
| El Platanillo | 16.95111 | -95.24417 | 346 | Ma | 10962 | M | 332 | 295 | 19 | 41.2 | NA | NA | NA | NA |
| El Platanillo | 16.95111 | -95.24417 | 346 | Ma | 10966 | J | 85 | NA | 0.5 | 13 | NA | NA | NA | NA |
| El Platanillo | 16.95111 | -95.24417 | 346 | Ma | 10968 | M | 290 | NA | NA | 31.7 | NA | 1 | NA | NA |
| El Platanillo | 16.95111 | -95.24417 | 346 | Ma | 10969 | M | 352 | NA | 16 | 41.2 | NA | 2 | NA | NA |
| Río Jaltepec | 17.38844 | -95.05611 | 40 | Ap | 209141 | M | 420 | 380 | 20 | 39.9 | NA | NA | 12 | NA |
| Río Jaltepec | 17.38844 | -95.05611 | 40 | Ap | 209158 | M | 490 | NA | 20 | 81.5 | NA | NA | 16 | NA |
| Río Jaltepec | 17.38844 | -95.05611 | 40 | Ap | 209162 | J | 240 | NA | 10 | 12.8 | NA | NA | NA | NA |
| Río Escondido | 17.09108 | -94.75169 | 83 | Ap | 200957 | M | 300 | NA | 13 | 31 | 1 | NA | 4 | NA |
| Río Escondido | 17.09108 | -94.75169 | 83 | Ap | 200958 | F | 310 | 270 | 25 | 35.4 | NA | NA | 1 | NA |
| Río Escondido | 17.09108 | -94.75169 | 83 | Ap | 20961 | M | 345 | 290 | 25 | 34.8 | NA | NA | NA | NA |
| Río Escondido | 17.09108 | -94.75169 | 83 | Ap | 20961bis | M | 195 | NA | 8 | 8.6 | NA | NA | NA | 2 |
| Río Escondido | 17.09108 | -94.75169 | 83 | Ap | 20964 | J | 215 | 186 | 10 | 11.3 | NA | NA | NA | NA |
| Río Escondido | 17.09108 | -94.75169 | 83 | Ap | 20965 | NA | 240 | NA | 11 | 17.4 | NA | NA | 1 | NA |
| Río Escondido | 17.09108 | -94.75169 | 83 | Ap | 20966 | F | 300 | NA | 15 | 36 | NA | NA | 4 | 1 |
